# Society for cardiovascular magnetic resonance expert consensus statement on quantitative myocardial perfusion cardiovascular magnetic resonance imaging^☆^

**DOI:** 10.1016/j.jocmr.2025.101940

**Published:** 2025-08-08

**Authors:** Amedeo Chiribiri, Andrew E. Arai, Edward DiBella, Li-Yueh Hsu, Masaki Ishida, Michael Jerosch-Herold, Sebastian Kozerke, Xenios Milidonis, Reza Nezafat, Sven Plein, Cian M. Scannell, Michael Salerno

**Affiliations:** a School of Biomedical Engineering and Imaging Sciences, King’s College London, London, United Kingdom; b National Institute for Health and Care Research (NIHR) Healthtech Research Centre (HRC) in Cardiovascular and Respiratory Medicine, Guy’s & St Thomas’ NHS Foundation Trust, London, United Kingdom; c Cardiovascular Medicine and Department of Radiology, University of Utah, Salt Lake City, Utah, USA; d Electrical and Computer Engineering Department, University of Utah, Salt Lake City, Utah, USA; e Department of Biomedical Engineering, University of Utah, Salt Lake City, Utah, USA; f National Heart, Lung and Blood Institute, National Institutes of Health, Bethesda, Maryland, USA; g Department of Radiology, Mie University Graduate School of Medicine, Tsu, Mie, Japan; h Cardiovascular Division and Department of Radiology, Brigham and Women's Hospital, Boston, Massachusetts, USA; i Institute for Biomedical Engineering, University and ETH Zurich, Zurich, Switzerland; j CYENS Centre of Excellence, Nicosia, Cyprus; k Department of Medicine, Cardiovascular Division, Beth Israel Deaconess Medical Center and Harvard Medical School, Boston, Massachusetts, USA; l Multidisciplinary Cardiovascular Research Centre, Department of Biomedical Imaging Science, Leeds Institute of Cardiovascular and Metabolic Medicine, University of Leeds, Leeds, United Kingdom; m Department of Biomedical Engineering, Eindhoven University of Technology, Eindhoven, Netherlands; n Department of Cardiovascular Medicine, Stanford University, Stanford, California, USA; o Department of Radiology, Cardiovascular Imaging, Stanford University, Stanford, California, USA

**Keywords:** Myocardial perfusion imaging, Coronary artery disease, INOCA, Kawasaki disease, Cardiovascular magnetic resonance, Stress perfusion, Quantitative perfusion

## Abstract

Myocardial perfusion imaging plays a central role in the management of patients with known or suspected coronary artery disease (CAD) and increasingly in patients with suspected ischemia with normal coronary arteries (INOCA) as well as anomalous origins of the coronary arteries and Kawasaki disease. Stress perfusion cardiovascular magnetic resonance (CMR) is recognized by international guidelines, with several Class 1 indications for the detection of abnormal myocardial blood flow in these clinical scenarios and offers excellent diagnostic accuracy and independent prognostic value. While visual interpretation of the perfusion data is the prevailing analysis method in clinical practice, quantitative perfusion CMR is at least as accurate for the detection of significant obstructive CAD and provides a more accurate estimation of the total ischemic burden in patients with CAD. Moreover, quantitative myocardial perfusion analysis provides unique insights into the pathophysiology of myocardial ischemia, including microvascular disease in INOCA. Quantitative perfusion CMR can be fully automated, is user-independent, and may facilitate more widespread use of the modality. The aim of this Society for Cardiovascular Magnetic Resonance (SCMR) expert consensus document is to provide recommendations for the acquisition and analysis of quantitative myocardial perfusion CMR to facilitate standardization of methodology. This paper also discusses research and development goals to address current limitations, to ensure data reliability and validity, to create the basis for future multi-vendor and multicenter research, and to broaden the clinical use of quantitative perfusion CMR.

## 1 Summary of acquisition and analysis recommendations

### 1.1 Image acquisition

• Given current evidence, two-dimensional (2D) acquisition is the recommended approach for quantitative perfusion cardiovascular magnetic resonance (CMR).
• A saturation-recovery dual-sequence with balanced steady-state free precession (bSSFP) or gradient echo (GRE) cartesian readout is recommended. At 3.0T, GRE readout is preferred, but bSSFP can be considered if off-resonance and frequency shift effects are taken into consideration and adequate modelling of T2 effects can be provided.
• Minimum spatial coverage is three slices in short-axis orientation (base, mid, apex). Minimum spatial resolution is 2.5 × 2.5 mm^2^ in-plane, with a maximum slice thickness of 10 mm. Target temporal acquisition window for the readout of the myocardial images of <120 ms to minimize motion and dark rim artefacts (DRA), and temporal resolution (i.e., image update rate) equivalent to 1 RR interval. Saturation time (TS) values for the myocardium should not exceed 130 ms.
• Saturation pre-pulse must have a high saturation efficiency (>95%). This can be achieved using non-slice-selective composite pulse trains or adiabatic pulses.
• GRE readout with linear ordering is recommended for arterial input function (AIF) acquisition. Maximum TS values for the AIF should be <30 ms.
• Proton density (PD)-weighted images should be acquired in the first 1-4 frames (for both the AIF and tissue function for coil sensitivity correction and/or conversion to T_1_ or contrast agent concentration).
• Free shallow breathing is recommended particularly when motion correction is available.
• A minimum number of time frames sufficient to acquire the complete first pass of the contrast agent should be recorded.
• At least the AIF, and ideally also the myocardium, should be sampled every heartbeat.
• Non-rigid motion correction should be available for perfusion quantification as part of the reconstruction or post-processing pipeline, both for the AIF and myocardial images. Motion correction should address alignment of PD-weighted images as well.
• A minimum dataset containing the following information should be exported in Digital Imaging and Communications in Medicine (DICOM) format from the scanner:
  ▪ Non-motion-corrected as well as motion-corrected dynamic myocardial images (including PD-weighted images).
  ▪ Non-motion-corrected as well as motion-corrected dynamic AIF images, when a dual-sequence technique is used (including PD-weighted images).
  ▪ The following sequence parameters for both the myocardial and AIF images: flip angle, repetition time (TR), echo time (TE), TS (as defined from saturation to the center of k-space), time delay (TD; defined as the delay from saturation to the first readout radiofrequency [RF] pulse), number of lines to center of k-space, and acquisition time and/or dynamic scan interval for each frame. These parameters, which are required for Bloch simulations, might not correspond to standard DICOM fields and might need to be conveyed through private tags.
  ▪ Type and dosage of contrast agent should also be noted.

### 1.2 Contrast agent dose and delivery regime

• Across multiple research studies and clinical practice single bolus injections with a dose of 0.05–0.075 mmol of Gd per kg of body weight have been used in combination with a dual-sequence acquisition (total gadolinium-based contrast agent [GBCA] dose 0.1–0.15 mmol of Gd/kg of body weight).
• In the United States, gadobutrol at a recommended total dose of 0.1 mmol/kg has been United States Food and Drug Administration (FDA) approved with a clinical indication of cardiac magnetic resonance imaging (MRI) to assess myocardial perfusion at stress and rest and late gadolinium enhancement in adult patients with known or suspected coronary artery disease. The protocol that was evaluated in the pivotal trial (GADACAD) utilized a dose of 0.05 mmol/kg at stress and rest with an injection rate of 4mL/sec.
• In routine clinical practice, a contrast dose in the range of 0.1–0.15 mmol/kg are recommended with consideration of regional regulatory guidances.
• Flow rates of 3-5 mL/s are recommended. Lower rates may be appropriate for smaller children and for higher relaxivity contrast agent to avoid saturation in the blood-pool compartment. The use of a relatively large vein (i.e., antecubital vein) is recommended, whenever technically possible.
• A dual-bolus technique is a possible alternative if a dual-sequence technique is not available. There is consensus, however, that a dual-sequence technique is preferred whenever available as it is more robust and easier to perform in routine clinical practice.

### 1.3 Stress agents

• The infusion of adenosine, adenosine triphosphate (ATP), dipyridamole or regadenoson can be utilized to accurately assess stress myocardial blood flow. When dipyridamole or regadenoson is used, these may preclude accurate estimation of MPR due to their relatively long half-life.
• Physiological responses to stress (e.g., heart rate, blood pressure, or clinical symptoms) should be monitored and recorded, and the dose increased if an adequate stress response is not observed. Splenic switch-off may not be a reliable indicator of stress response. Stress imaging should be performed first. A sufficient time between stress and rest images (≥10 min) should be allowed for the patient return to a resting state.
• For a stress-rest protocol (stress performed first) adenosine is recommended as it provides accurate assessment of both stress and rest myocardial blood flow leading to more accurate assessment of myocardial perfusion reserve (MPR).

### 1.4 Image pre-processing

• Motion correction significantly improves the quality of the perfusion maps and should be used.
• Correction for coil sensitivity and/or conversion of image signal intensity (SI) to T_1_ or contrast agent concentration is essential for quantification. When converting to T_1_/contrast agent concentration, a Bloch-equation conversion method is recommended for quantification of dual-sequence data [1]. Conversion to T_1_/contrast agent concentration may not be required when alternative quantification methods based on transit-time estimation are used or other approaches are adopted to limit the non-linearity between contrast concentration and image SI.
• Conversion of SI to T_1_/contrast agent concentration requires correction for baseline T_1_. A fixed T_1_ value suitable for the specific patient population and scanner is preferred over patient-specific T_1_ values to facilitate processing. Baseline correction should be applied to the SI-time or T_1_/contrast agent concentration curves.
• Spatial filtering should be limited to the high-resolution myocardial slices (including PD-weighted images) and should be kept to a minimum or avoided if the signal-to-noise ratio (SNR) is sufficient for quantification. Temporal filtering is not recommended as image pre-processing step.
• LV blood-pool detection may be performed using any method that ensures exclusion of the papillary muscles and endocardial partial volume effects. A machine learning approach is recommended for automated detection.
• Myocardial segmentation may be performed either on the perfusion images before quantification or on the myocardial blood flow (MBF) maps following quantification and should be devoid of blood pool or extracardiac tissue contamination. Machine learning is recommended for automated segmentation.

### 1.5 Quantification methods

Pixel-wise quantification is recommended to preserve the high spatial resolution of myocardial perfusion CMR. The following approaches have been adopted previously and validated.

Deconvolution (e.g., Fermi-function deconvolution):

o It is essential to use the first-pass component of the SI-time curves.
o Including a parameter for interstitial effects is reasonable at the expense of an additional modelling parameter.

Tracer-kinetic models:

o Allow modelling the SI-time response beyond the first pass of the contrast agent. These models typically have more parameters but are more physiologically plausible.

Parsimonious models using fewer parameters would be preferable, reducing the inherent uncertainties secondary to the use of assumed parameters derived from basic science experiments. Least-squares parameter estimation is recommended with a validated selection of fitting algorithms and settings to ensure the accuracy and repeatability of quantitative values.

### 1.6 Interpretation

• Cut-off values for abnormal MBF and MPR vary across implementations and need to be standardized. Exact thresholds for a normal MPR are not universally accepted at this time but are generally on the order of an MPR of >2.
• Significant differences in MBF values have been demonstrated by CMR and positron emission tomography (PET) between male and female subjects [2], [3]. These differences may also affect MPR values and should therefore be considered when interpreting quantitative perfusion maps.
• Potential artifacts need to be considered within the context of both quantitative stress and rest perfusion images and maps. Perfusion images and maps should be reviewed side-by-side or superimposed with the ability to quickly change from quantitative map to perfusion images.
• Pixel-wise perfusion maps should be related to coronary perfusion territories using the American Heart Association (AHA) 16-segment model as guidance. Patient-specific anatomical variations should be considered when assigning AHA segments to coronary territories.
• Perfusion defects should be quantified in terms of percent of myocardium affected rather than number of segments affected.
• Quantitative perfusion maps showing a uniform increase in MBF from rest to stress or a significant MPR are excellent quality assurance metrics to confirm an adequate vasodilator response and a true negative stress perfusion test.

### 1.7 Quality control

The following recommendations are aimed at advising users on how to detect potential issues arising in the described steps to generate quantitative perfusion maps:

• Raw and motion-corrected perfusion images should also be reviewed qualitatively along with the quantitative perfusion maps.
• Plots of the RR interval should be reviewed, looking for regular rhythm versus irregular rhythm or missed beats. Missed beats during the upslope of the AIF or the upslope of the myocardial SI-time curve could corrupt quantification [4].
• Be cautious of measurements in cases where the quantitative map either looks different in shape or wall thickness than the raw images. This may be particularly relevant in apical slices due to partial volume effects.
• It is recommended that a map of myocardial tracer arrival times be produced as part of the quantification output and examined for quality control. MBF estimates are sensitive to the estimates of tracer arrival times, and tracer arrival may be a useful parameter, e.g. as marker of collateral-dependent myocardium [5], [6]
• Automated endocardial and epicardial contours should be verified.
• Verify that the AIF region of interest is in the left ventricular (LV) cavity or aortic root as expected for the quantification software being used and excludes papillary muscles and endocardial partial volume effects.
• Myocardial SI-time curves and the detected tracer-arrival times should be visually reviewed whenever possible with a resolution not inferior to that of standard AHA segments.
• AIF curves should be reviewed qualitatively. A smooth AIF is indicative of sufficient motion correction and high SNR.

## 2 Introduction

Myocardial perfusion cardiovascular magnetic resonance (CMR) imaging has become an established diagnostic test for the identification of myocardial ischemia based on large multicenter multivendor trials [7], [8], [9], [10], [11], which provide the foundation for high-level indications in clinical practice guidelines [12], [13]. Perfusion CMR images are usually analyzed by qualitative visual assessment. This approach relies on the identification of areas of myocardium with relatively delayed and reduced dynamic contrast enhancement during the first passage of a bolus of a gadolinium-based contrast agent through the myocardium [14]. Visual analysis of myocardial perfusion CMR has been validated against invasive coronary angiography [15] and fractional flow reserve (FFR) [16], with excellent diagnostic accuracy for the detection of obstructive coronary artery disease (CAD), which has been shown to be superior to nuclear imaging methods in large multicenter multivendor trials [7], [10], [11], [17]. Several studies have also demonstrated the strong and independent prognostic value [18], [19] and cost-effectiveness [20], [21], [22], [23], [24] of stress perfusion CMR. International practice guidelines attribute stress perfusion CMR several Class 1 indications, at similar levels to the other non-invasive cardiovascular imaging methods [12], [13], [25].

Pediatric clinical applications include the assessment of anomalous origins of the coronary arteries, coronary artery fistulae, as well as status post coronary artery reimplantation, iatrogenic injury during congenital heart surgery, and Kawasaki disease [26], [27], [28], [29].

Quantitative myocardial perfusion analysis has been proposed as an addition or alternative to visual assessment. By enabling the detection and quantification of myocardial blood flow (MBF) in absolute terms, it offers the potential for decision-making with minimal or no observer dependence, and standardized thresholds for determining ischemic burden to guide revascularization. However, stress perfusion CMR with visual analysis is currently one of the most accurate and reliable methods for assessing myocardial ischemia, as demonstrated by multiple studies [7], [8], [9], [10], [11]. Therefore, for quantitative myocardial perfusion CMR to replace visual analysis, it must first achieve the highest levels of scientific evidence to justify its recommendation for routine clinical use. This position paper aims to provide the technical basis for quantitative myocardial perfusion analysis, standardizing methodology to foster multivendor and multicenter trials, and future clinical adoption. Quantitative myocardial perfusion CMR enables the derivation of measurements of myocardial perfusion (or MBF) in units of [mL/min/g of tissue] and myocardial perfusion reserve (MPR, defined as the ratio between stress and rest MBF values) from perfusion CMR series [30], [31], [32], [33]. Quantitative analysis offers several potential advantages over qualitative visual assessment. It is superior to visual assessment in the detection of multivessel CAD and in the quantification of the total ischemic burden [34], [35], [36]; it can detect and quantify globally reduced MBF, as frequently observed in patients with coronary microvascular disease [37], [38], [39], [40]; it allows the definition of objective and reproducible thresholds of myocardial ischemia and ischemic burden for the monitoring of disease progression and assessment of the efficacy of treatments [2], [41]; it has good agreement with microsphere measurements [42], [43], [44], [45], [46], positron emission tomography (PET) [47], [48], [49], [50] and invasive indices of myocardial perfusion [37], [51], [52]; and it has prognostic relevance in patients with CAD [53], [54]. Additionally, quantitative myocardial perfusion CMR may play a role in assessing patients with non-ischemic cardiomyopathies [55], [56], [57], [58], [59] and a range of other and less common clinical scenarios [60], [61], [62], [63].

The recent availability of investigational software tools for fully automated analysis of quantitative myocardial perfusion has transformed quantification from a time-consuming image-processing technique, previously available only to a few specialist centers worldwide, to an almost real-time process, potentially available to most clinical centers with a wide range of expertise [64], [65], [66]. However, for quantitative myocardial perfusion CMR to become a clinically indicated tool, it requires standardization of methods and validation in large multicenter, multivendor clinical studies. Moreover, it is essential that the acquisition sequences, contrast agents and analysis tools used for quantitative perfusion analysis receive regulatory approval for this application. As of the publication of this consensus document, no commercially available software packages for quantitative perfusion analysis exist, limiting its clinical use.

The aim of this Society for Cardiovascular Magnetic Resonance (SCMR) expert consensus statement is to summarize the current methods for the acquisition and analysis of dynamic first-pass quantitative myocardial perfusion CMR, and to give recommendations for best practices. Specifically, this document focuses on image acquisition, image processing and on methods for quantitative analysis. It also discusses research and development goals to address current limitations, to ensure data reliability and validity, and standardizing methodology, to create the basis for future multivendor and multicenter trials, aiming at the eventual clinical adoption. This consensus statement is based on published data and on the shared experience of the members of the expert panel. Other CMR techniques for the identification of myocardial ischemia, such as blood oxygen level dependent (BOLD) imaging [67], arterial spin labelling (ASL) [68], stress and rest phase-sensitive flow measurements in the coronary sinus [69], [70], as well as other analysis methods (e.g., semi-quantitative perfusion analysis) are beyond the scope of this consensus statement. The document does not cover the indications or clinical use of quantitative myocardial perfusion CMR, which will be the subject of a future SCMR consensus document.

## 3 Methodology

The SCMR has constituted a Special Interest Group on the topic of quantitative perfusion CMR, which consists of medical physicists, physicians, and biomedical engineers, with the aim to discuss the state of the technique and directions for future work. The SCMR Special Interest Group has identified the need for the current document as an essential step towards the clinical adoption of quantitative perfusion CMR.

The expert panel and writing group for this document were formed following guidance of the SCMR Executive Committee and membership was approved by the SCMR Board of Trustees. The expert panel and writing group included cardiologists, radiologists, and basic scientists with recognized expertise in quantitative perfusion CMR from diverse geographical locations. The writing group reviewed the literature and prepared a manuscript draft which was reviewed and edited by the expert panel. Each recommendation was discussed and agreed by majority. The document was then reviewed by an external group of experts in the field of CMR under the leadership of the SCMR Publications Committee and of the SCMR Board of Trustees.

### 3.1 Image acquisition

#### 3.1.1 General

Dynamic first-pass myocardial perfusion CMR visualizes the passage of a bolus of gadolinium-based contrast agent (GBCA) through the heart and the myocardium. Each slice of the myocardium is imaged sequentially every heartbeat throughout the dynamic acquisition using electrocardiography or other types of gating. All dynamics of each slice are imaged in the same phase of the cardiac cycle in consecutive heart beats. The planning of quantitative perfusion acquisitions does not differ from that of standard perfusion sequences for visual assessment and should be performed according to the recommendations of current SCMR acquisition guidelines [71]. At least three short-axis images should be acquired every RR interval at basal, mid-ventricular and apical level. Additional short-axis or orthogonal long-axis images may be acquired if the pulse sequence used allows for this with preserved spatial and temporal resolution.

For quantitative perfusion analysis, acquisition at alternate heart beats to accommodate additional slices is not recommended. Data acquisition typically extends over 40–60 heart beats, timed to coincide with the cardiac passage of an intravenously injected bolus of contrast agent. The acquisition of a higher number of frames might be required in the setting of a stress heart rate >100 beats per minute, especially in subjects with low ejection fraction. As general guidance, the acquisition should include a number of frames sufficient to visualize at least the full first pass of the bolus of contrast agent through the myocardium.

#### 3.1.2 Patient preparation

The preparation of the patient and contraindications to the scan do not differ from a standard stress perfusion CMR scan [71]. Young children and infants typically need to be scanned under general anesthesia or sedation [72]. Specific precautions apply to examinations in sedated or anaesthetized patients because of their inability to report chest pain and other discomforts and side effects [73]. The use of a power injector is a pre-requisite for quantitative perfusion acquisitions. When adenosine, dipyridamole or adenosine triphosphate (ATP) infusion is used, it is recommended to cannulate two veins, one in each arm, ideally in the anterior cubital fossa. One cannula is used for the injection of the stress agent and one for the injection of the GBCA. The cannula used for contrast injection should ideally allow for a power-injection at 3–5 mL/s. With smaller bore catheters the flow may need to be reduced depending on the cannula specifications, but should ideally be at least 3 mL/s, or 2 mL/s in younger children. When regadenoson is used as stress agents, a single intravenous cannula is sufficient. If it is only possible to cannulate one vein, the use of this drug should therefore be considered, if locally available.

Blood pressure should be monitored before, during and after administration of the stress agent to gauge the response to the stressor. When continuous infusion of the stress agent is required, the blood pressure cuff used for monitoring should be positioned on the arm in which gadolinium contrast agent will be injected. The blood pressure cuff should not be positioned on the arm in which the continuous infusion of the stress agent is performed. Care should be taken to make sure that the blood pressure cuff is not inflated at the time of the contrast administration.

#### 3.1.3 Pulse sequences

Spatial and temporal resolution, spatial coverage and robustness against motion and other artifacts are parameters of general concern in every image acquisition. Contrast-enhanced quantitative perfusion imaging additionally requires a known relationship between the signal and T_1_ changes as induced by the contrast agent bolus:

(1) $$\frac{1}{T_{1}}=\frac{1}{T_{1,0}}+r_{1}∙\left[\mathrm{CA}\right]$$

Here, $T_{1,0}$ and $r_{1}$ denote baseline (native)$\;T_{1}\;$of the tissue and relaxivity and (CA) the concentration of the contrast agent, respectively. In principle, the T_1_ dependency of driven steady-states may be exploited to deduce contrast agent concentration [74], [75]. However, the time to steady-state often renders this approach problematic. Magnetization preparation schemes either using inversion recovery or saturation recovery (SR) pre-pulses for $T_{1}$weighting have been tested for perfusion imaging. SR has been most widely adopted due to its robustness to mis-triggering, heart rate variation and motion during the scan as the magnetization is “reset” with each SR preparation. In a SR sequence, a carefully calibrated 90° saturation preparation pulse is used to minimize the pre-contrast signal of the myocardium and of the left ventricular (LV) blood pool and to hence visualize the passage of the contrast bolus, generating images with strong $T_{1}$-weighting. SR preparation is the approach recommended for perfusion imaging, including quantitative perfusion CMR.

**Fig. 1:**
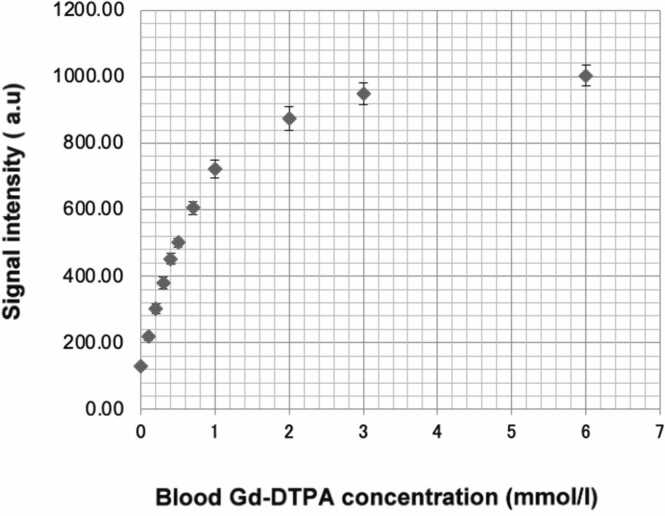
Blood SI vs Gd-DTPA concentration. Data derived from 11 tubes containing blood samples from 5 healthy volunteers with different concentrations of Gd-DTPA (0, 0.1, 0.2, 0.3, 0.4, 0.5, 0.7, 1.0, 2.0, 3.0, and 6.0 mmol/liter; 9 mL of blood and 1 mL of blood or diluted Gd-DTPA) were acquired for each subject using a saturation-recovery bSSFP sequence (TR = 3.0 ms, TE = 1.5 ms, flip angle = 40°, FOV = 36 × 32 cm2, section thickness = 8 mm, acquisition matrix size = 192 × 154, SENSE factor = 2, time between saturation preparation pulse and center of k-space acquisition = 200 ms, and duration of image data acquisition = 210 ms). Imaging of the blood tubes was repeated five times for blood samples and was completed within 1 h after the blood was taken from each volunteer. Regions of interest were drawn inside the tubes, and the mean SI was measured to obtain the average curve of the blood SI vs. blood Gd-DTPA concentration for the five subjects. *Gd-DTPA* Gadolinium-Diethylene-Triamine-Pentacetic acid, *TR* repetition time, *TE* echo-time.

Reproduced with permission from Ichihara et al [105].

Any saturation strategy used for quantitative perfusion imaging must have high saturation efficiency (>95%). Given the presence of both B_0_ and B_1_ inhomogeneities, composite pulse trains or adiabatic pre-pulses are typically utilized [76], [77]. Importantly, the relationship between signal and contrast agent concentration can only be approximated as linear for [78]: (1) very short delays between saturation pre-pulse and acquisition of the center of k-space, also known as saturation time (TS), in case of high contrast agent concentration, or for: (2) low contrast agent concentrations (Fig. 1). Knowledge of the TS and other parameters is required for obtaining the correct conversion from magnetic resonance signal to contrast agent concentration.

The visualization of three to four slices in each cardiac cycle can be achieved using either gradient-echo (GRE) acquisitions [74], [79], [80], gradient echo-planar imaging (GRE-EPI) [81], [82], [83] or balanced steady-state free precession (bSSFP) pulse sequences, and demands very rapid data acquisition [75], [84]. For 2D acquisitions, a desirable temporal acquisition window for each image is <120 ms and optimally <100 ms. TS values for the myocardium should not exceed 130 ms. These requirements inherently limit spatial resolution and coverage that can be obtained with Cartesian acquisitions and with conventional parallel imaging techniques. For example, for a 100 ms acquisition window per slice in each cardiac cycle, a repetition time (TR) of 2.5 ms, 80% partial Cartesian sampling, parallel imaging with 2-fold undersampling and a field of view (FOV) of 300 mm, the achievable in-plane resolution is 3 mm. At this resolution, subendocardial perfusion defects may be difficult to discern, and dark rim artifacts (DRA) can be problematic.

Some technical developments have been proposed to increase spatiotemporal resolution even further by jointly exploiting compressed sensing (CS) and parallel imaging [85], by radial and spiral trajectories in conjunction with simultaneous multi slice (SMS) [86], [87], [88], [89], [90], *k-t* undersampling [91], [92], [93] by using localized spatiotemporal constraints [94], or deep-learning (DL) approaches [95], [96], [97]. Another avenue of development concerns novel approaches to steady-state imaging without magnetization preparation [98], [99], simplifying patient setup and increasing robustness against arrhythmias. One key consideration with non-cartesian, low-rank, CS, or DL reconstructions is to ensure that the process does not alter significantly the temporal time course of the first-pass signal response, as this will propagate into the quantitative metrics.

There is also a continuing interest in increasing heart coverage either by 2D approaches or 3D approaches [100], [101], [102]. Limited data are available at the current point in time as to whether the increased coverage improves diagnostic performance. There is, however, consensus that this could lead to a more accurate assessment of the ischemic burden. Though whole-heart coverage is desirable, data regarding the use of 3D acquisitions for quantitative analysis are limited, the required sequences are not widely available and most quantitative analysis pipelines have been developed, tested, and validated with 2D data. A move from 2D to 3D represents a challenge for established analysis tools to account for the differences in the input data, and new approaches would need to be considered for steps such as motion correction. 3D acquisition methods are, therefore, not recommended at this stage for quantitative perfusion analysis.

Based on these considerations, for quantitative perfusion analysis, the current recommendation for image acquisition is to use 2D multi-slice sequences to obtain at least three short-axis slices, ideally with one RR temporal resolution and an acquisition window of <120 ms. T1-weighting should be identical for all slices by repeating the SR preparation for each slice and using non-slice-selective preparations to avoid alteration of T1-weighting caused by through-plane motion. No recommendation can be given on the acceleration schemes to achieve these parameters, other than to ensure that the reconstruction preserves the temporal fidelity of the contrast agent dynamics. This is less of a concern with frame-by-frame conventional parallel imaging.

#### 3.1.4 Arterial input function

Quantitative perfusion CMR analysis requires knowledge of both the myocardial tissue response and the arterial input function (AIF), i.e., the signal intensity (SI) time curve acquired in the blood pool (typically the LV). A linear relationship between signal and contrast agent concentration is desirable for quantitative perfusion analysis [31]. Linearity is approximated at low contrast agent concentrations. This may, however, reduce the contrast-to-noise ratio (CNR) between non-ischemic and ischemic myocardium and hence compromise the qualitative reading of the myocardial signal response. On the other hand, at contrast agent dosages between 0.05 and 0.1 mmol of GBCA/kg of body weight, as used clinically, there is a significant departure from linearity between measured AIF signal and contrast agent concentration (Fig. 1) [103], [104], [105].

**Fig. 2:**
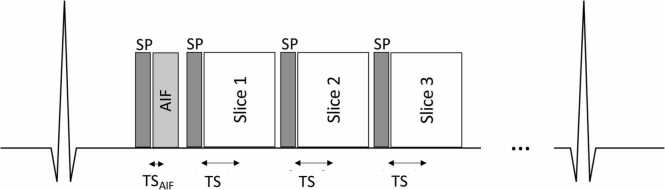
Dual-sequence technique including a module to image the AIF with a short delay between SP and k-space center followed by the standard perfusion imaging module (TS, saturation time; TS_AIF_, saturation time of the arterial input function). All saturation times (TS and TS_AIF_) are measured from the time of saturation SP to the center of k-space of each slice. *AIF* arterial input function, *SP* saturation pre-pulse.

The differences in contrast concentration in the myocardium and the blood pool require a dedicated approach to acquiring the myocardial tissue response and the AIF to ensure linearity. To capture the nonlinear signal/concentration relationship, modifications to the GBCA injection scheme or to the pulse sequence have been proposed. In the *dual-bolus technique* [1], [43], [106], [107], [108], a diluted GBCA bolus is used for AIF imaging, followed by a full concentration bolus for the myocardial perfusion images. Alternatively, the *dual-sequence technique* [109] interleaves the acquisition of the myocardial slices with a second sequence for sampling the AIF which has low spatial resolution and significantly shorter TS (<30 ms), thereby reducing the non-linearity of the signal response (Fig. 2).

For the AIF acquisition in a dual-sequence regime, a GRE readout with linear ordering is recommended. If the heart rate increase during vasodilatory stimulation exceeds the maximum rate that permits acquisition of each slice and the AIF in every heartbeat, it is recommended to prioritize acquiring the AIF at every heartbeat while acquiring myocardial slices on alternate beats. This may require a specialized pulse sequence.

The dual-sequence has several advantages over the dual-bolus technique as it does not require dilution of the contrast agent and the complex setup of the injection lines for two separate bolus injections. The dual-sequence technique enables direct injection of a single bolus of undiluted gadolinium contrast agent with a power injector, as used in current clinical routine for visual assessment.

Recently, DL approaches such as the use of convolutional neural networks have also been proposed to model the nonlinear relationship between signal and CA concentration when a dual-sequence implementation is not available [110]. However, more validation of this approach is required before a recommendation for this method can be given.

The recommendations assume sampling of the AIF in the LV. However, the AIF can also be sampled in locations other than the LV (i.e., ascending aorta), depending on the specific sequence implementation [111], [112]. It is recommended that the AIF should be sampled near the base of the LV (i.e., basal slice) or should incorporate the aortic root.

From a practical standpoint, the dual-sequence technique is recommended—when available—due to its ease of use in clinical settings. However, it is important to note that this method is not yet supported across all scanner platforms. The dual-bolus technique can therefore be a valid alternative when a dual-sequence is unavailable.

#### 3.1.5 Proton-density images

Perfusion CMR images are typically acquired with multi-element surface coil arrays to boost the signal-to-noise ratio (SNR), which can lead to spatial variation in SI across the FOV, termed coil sensitivity or coil bias. These inhomogeneities cause variations in the quantitative values and affect the ability to accurately quantify MBF [113]. The acquisition of proton density (PD) weighted images is recommended for the purpose of coil bias correction for quantitative perfusion imaging. PD-weighted images, typically acquired without magnetization preparation and using a low flip angle, are used during image post-processing for signal normalization to accurately quantify dynamic T_1_ changes during first pass.

#### 3.1.6 Data export

To allow for subsequent post-processing and quality control, data should be exported in digital image and communication in medicine (DICOM) format from the MRI scanner. Exported data should include at a minimum the non-motion-corrected as well as motion-corrected dynamic myocardial images (including PD-weighted images), and the non-motion-corrected as well as motion-corrected dynamic AIF images (including PD-weighted images) when a dual-sequence technique is used. In addition, DICOM headers should include the following minimum set of sequence parameters for both the myocardial and AIF images: flip angle, TR, echo time (TE), TS, number of lines to center of k-space, acquisition time and/or dynamic scan interval for each frame. TS should be defined from the time of saturation to the time of acquisition of the center line of k-space (Fig. 2). In addition, the time delay (TD) between the time of the saturation and the time of the first readout radiofrequency (RF) pulse center should also be specified. These parameters, which are required for Bloch simulations, might not correspond to standard DICOM fields and might need to be conveyed through private tags.

### 3.2 Contrast agent dose and delivery regime

From a clinical and technical perspective, gadoteric acid, gadoteridol, and gadobutrol can be used interchangeably for quantitative stress perfusion imaging. Local availability and regulatory approval may differ from one region to another. For example, in the US gadobutrol is the only FDA-approved agent for the indication of myocardial stress and rest myocardial perfusion imaging and late gadolinium enhancement in patients with known or suspected CAD, with a suggested total dose of 0.1 mmol/kg of body weight [114]. The pivotal GADACAD trial used 0.05 mmol/kg at stress and rest with an injection rate of 4 mL/s for stress and for rest [115]. The type of contrast agent used should be noted, as this may need to be considered during the quantification process, depending on the method used. A dose of 0.05–0.075 mmol of GBCA per kg of body weight has been used in clinical studies and, subject to local regulatory approval, is recommended for a dual-sequence technique [115], [116], injected preferably at 3–5 mL/s, followed by a saline flush of 20–25 mL injected at the same rate as the bolus of contrast agent. If a dual-bolus technique is used, it is recommended to use a 10% dilute contrast pre-bolus of the same volume as the main bolus [108]. It is important to note, however, that this is a clinical recommendation which might not align with the regulatory approved use of the GBCA in use in some regions. Both the pre-bolus and main bolus should be injected at 3–5 mL/s and be followed by a saline flush of 20–25 mL injected at the same rate as the bolus of contrast agent. Pre-bolus and main bolus should be separated by at least 25 s, in particular for rest acquisitions and in patients with low cardiac output [108]. A relatively large vein (i.e., cubital vein) is the preferred site of injection as it allows faster injection rates, which produce a more compact bolus of contrast agent.

### 3.3 Stress agents

Quantitative perfusion analysis and visual assessment are usually performed on the same CMR dataset. Therefore, the same pharmacological stress regimes that are recommended for visual perfusion analysis should also be used for quantitative perfusion CMR [71]. Vasodilators (i.e., adenosine, dipyridamole, regadenoson, or ATP) are preferred over beta-adrenergic agents (i.e., dobutamine) for quantitative stress perfusion CMR due to a better safety profile and lower heart rate at peak stress [117], [118]. Most published studies have used adenosine for vasodilator stress perfusion CMR. Only a few studies have compared different vasodilatory stress agents in the setting of quantitative perfusion analysis, suggesting that stress MBF values derived from regadenoson, dipyridamole, and adenosine stress are broadly comparable [119], [120]. However, MPR may be underestimated when long-acting agents such as regadenoson and dipyridamole are used and stress images are acquired before recovery images. This is particularly important when assessing for possible microvascular disease, which can be characterized by a diffuse reduction in MPR values, and particularly in the subendocardial layer [121]. Reversal of the vasodilatory effects of the stress agent with aminophylline and a delay of at least 15 min between stress and rest imaging may mitigate this limitation. However, a previous study suggested an incomplete reversal of the effects of regadenoson using this approach [119]. For adenosine and ATP a minimum 10-minute interval between stress and rest imaging is recommended.

In awake pediatric patients, the possibility of an inability to comply with the remainder of the examination following the exposure to a vasodilatory stressor must be considered. This and reports of an incomplete reversal of the effects of regadenoson with aminophylline [119] have led some pediatric centers to perform the resting perfusion studies first and the imaging during stress towards the end of the scan protocol.

For regadenoson and dipyridamole, fixed-dose regimes are used in adults. In children, regadenoson is most commonly dosed at 6–8 mcg/kg, up to the typical adult dose of 400 mcg [122]. Adenosine and ATP should be used at a starting dose of 140 mcg/kg of body weight/min and increased up to 210 mcg/kg of body weight/min if there is inadequate stress response (heart rate increase less than 10 bpm or systolic blood pressure decrease less than 10 mmHg or absence of clinical symptoms) [71], [123]. Splenic switch-off is not recommended as a reliable indicator of stress response, as it is not usually measured during the stress administration before perfusion data acquisition.

Based on these considerations, adenosine stress is recommended whenever locally available, particularly when MPR measurements are required. Similarly to visual perfusion protocols [71], an escalating dose regime tailored to clinical response currently has the largest body of evidence.

### 3.4 Image processing

**Fig. 3:**
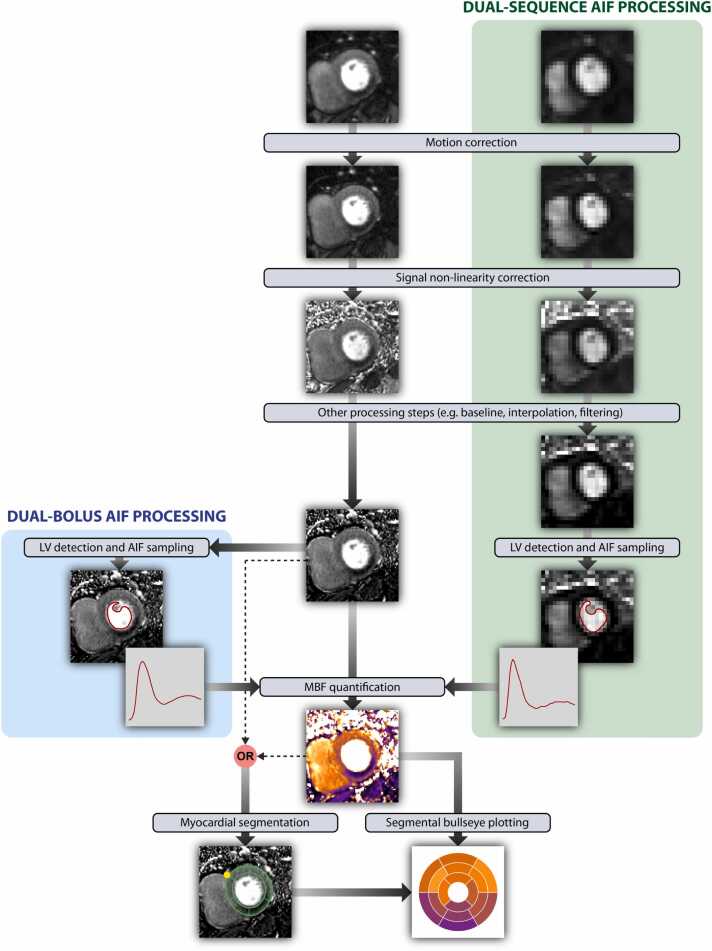
Workflow for first-pass myocardial perfusion imaging data processing and quantitative analysis. Starting from the raw perfusion images, the central column demonstrates the process followed for the analysis of high-resolution myocardial slices, while additional analyses aiming to sample the AIF for dual-sequence (green box) and dual-bolus techniques (cyan box) are also shown. Please note the low-resolution AIF acquired as an additional image when the dual-sequence approach is used. *AIF* arterial input function, *LV* left ventricle, *MBF* myocardial blood flow.

Quantification of perfusion at the pixel level necessitates the preparation of perfusion images with a series of processing steps. The overall aim is to alleviate the effect of physiological and imaging confounders that could compromise the accuracy and precision of MBF quantification. To standardize the quantification process, we propose the structured workflow shown in Fig. 3. The consensus within the field is to prioritize pixel-wise quantitative CMR processing, as illustrated in Fig. 3, as opposed to segment-wise quantification. This approach allows preservation of the high-resolution spatial information which is one of the key distinguishing features of stress perfusion CMR compared with other ischemia tests. Table 1 summarizes these image-processing steps and their impact on the accuracy and precision of quantitative perfusion measurements.

**Table 1:** Applications of different processing steps and impact on perfusion measurements.

Image processing step	Recommended for			Impact on perfusion values	
	Dual-bolus AIF images	Dual-sequence AIF images	Myocardial images	Accuracy	Precision
Motion correction	✓	✓	✓	++	++
Coil sensitivity correction	✗	✗	✓	++	+
Signal non-linearity correction	○	✓	○Dual bolus ✓Dual sequence	+++	-
T2* correction	✗	○	✗	+	-
Baseline correction	✓	✓	✓	++	-
Spatial filtering	✗	✗	○	-	+

*AIF* arterial input function. Please refer to the text for considerations regarding optional processing steps. (✓ recommended; ○ optional; ✗ not recommended; +++ very strong effect; ++ significant effect; + some effect; - no effect)

#### 3.4.1 Motion correction

One of the most important sources of error in quantitative analysis is motion-related artifacts arising from cardiac and respiratory motion, hampering both qualitative and quantitative analysis at the pixel level. While cardiac motion is often adequately addressed by prospective ECG-triggering, which allows the acquisition of each frame at the same cardiac phase, respiratory motion is often problematic. A simple method for alleviating respiratory motion is image acquisition during a breath-hold lasting at least through the first pass of the contrast agent. The disadvantage of this technique is that patients often have difficulty performing a long breath-hold during vasodilatory stress, and resumption of breathing during the first pass frequently results in significant through-plane motion that cannot be adequately corrected. Additionally, cardiac motion may still occur in patients with arrhythmias or due to triggering errors, even with prospective ECG-gating. Alternatively, free-breathing techniques are better suited for patients with difficulty breath-holding or complying with instructions. However, these require image processing routines, such as slice tracking or in-plane motion correction, performed either inline or offline, to estimate and compensate for respiratory motion.

Motion correction schemes based on image registration are commonly used to compensate in-plane inter-frame misalignment. However, applying general image registration algorithms to perfusion CMR images is challenging due to the dynamic contrast enhancement. Typical cost functions used in image registration often fail to disentangle the dissimilarity between consecutive time frames caused by inter-frame misalignment from those arising due to contrast agent dynamics. Motion correction can be achieved by exploiting the quasiperiodicity of respiratory motion [124] or by using methods based on optical flow estimation, without explicitly accounting for the contrast enhancement [125]. Recent work has focused on creating synthetic images for registration by either removing the contrast enhancement or iteratively removing motion [66], [88], [126], [127]. Prospective motion correction of the perfusion series has also been proposed [128]. It is also essential for motion correction to account for the alignment of PD-weighted images to the perfusion series.

The current recommendation is to use free-breathing sequences with integrated motion correction whenever available. Breath-hold acquisitions can be a valid alternative if motion correction techniques are unavailable. In these cases, operator assessment of the quality of the breath-hold and subsequent image registration is recommended. It remains uncertain whether motion-corrected spatial-temporal reconstruction or highly parallel acquisition followed by motion correction may provide the most optimal results.

#### 3.4.2 Coil sensitivity correction

Coil sensitivity correction techniques based on the acquisition and post-processing of PD-weighted images can be used to mitigate coil bias [113]. This correction typically involves fitting a polynomial surface, typically of quadratic form, to the PD-weighted images to estimate the coil bias field, which can subsequently be used to normalize the perfusion images. The intensity surface fitting approach has the advantage of providing a more homogenous SI gradient, but it requires careful detection of the myocardial region for accurate fitting.

Alternatively, when motion correction effectively aligns PD-weighted and perfusion images, a simpler approach consists in the pixel-wise normalization of perfusion images directly using the PD-weighted images. This approach is less affected by abrupt bright or dark regions in the PD-weighted images that tend to impact the reliability of surface fitting.

The current recommendation is to acquire PD-weighted images during the first few frames of both myocardial and AIF acquisitions for coil sensitivity correction and/or signal non-linearity correction (see *Signal non-linearity correction* section below).

#### 3.4.3 Signal non-linearity correction

Due to contrast mechanisms underlying CMR imaging, the SI recorded in the images has a nonlinear relationship to the concentration of the contrast agent [105]. To ensure myocardial perfusion is accurately measured, SI needs to be converted to contrast agent concentration. This conversion may be omitted in dual-bolus techniques acquiring both the arterial and myocardial signals using the same sequence parameters and at a correspondingly low concentration, with a reduced effect on linearity. However, conversion is imperative in dual-sequence techniques using a single high-concentration contrast bolus, as it accounts for the differences in saturation and T_1_ sensitivity of the AIF and myocardial readouts [129].

MRI does not directly measure the concentration of the contrast agent in tissue but instead detects its effect on nearby water molecules or water in exchange with the contrast-agent-permeated space. In practice, this means that, in myocardial tissue, the linear relationship between R_1_ and contrast concentration (Eq. 1) only holds at lower contrast concentrations, such that the rate of exchange of water between spaces with and without contrast is sufficiently fast to effectively result in a single exponential T1 relaxation. This is further reason not to exceed the recommended contrast agent dosages for quantitative perfusion imaging.

The extent of signal non-linearity depends on the pulse sequence parameters and the properties of the contrast agent. The process starts by converting SI to T_1_ relaxation times using a sequence-dependent equation or Bloch equation-based simulations. The estimated T_1_ is then inserted into Eq. 1 to yield the concentration of contrast agent. This calculation requires a baseline (native) T_1,0_, which can be a fixed or a patient-specific value measured using T_1_ mapping prior to each perfusion scan. Since both are considered equally appropriate choices, a fixed T_1,0_ suitable for the specific patient population and scanner is recommended to simplify the protocol.

The signal-to-T_1_ conversion approach can vary greatly depending on different sequence designs. While some make assumptions for simplification or use look-up tables with pre-determined combinations of SI and R_1_ or contrast agent concentration to speed up the conversion, signal dictionaries created using Bloch simulations are more suitable, in particular for bSSFP pulse sequences, where the signal-to-T_1_ relationship is more complex, due to the combined T_1_ and T_2_ weighting of the images.

The current recommendation is that conversion of SI to contrast agent concentration should always be applied when a dual-sequence technique is used, and should be considered when a dual-bolus technique is used. The conversion approach should be carefully implemented based on the type of readout and sequence design and must take into consideration the relaxivity of the GBCA used.

#### 3.4.4 T2* correction

At high concentrations of contrast agent, as observed in the LV during the first pass and reflected in the AIF, there is a further signal loss due to T_2_* effects. The T_2_* dephasing leads to an underestimation of the AIF peak and affects the accuracy of MBF measurements. It has been proposed [130], [131] to acquire multiple echo images with varying TE and fit the signal decay to the following equation:

(2) $$S(t)=S_{0}{(t)e}^{-\frac{\mathrm{TE}}{{T2}^{*}(t)}}$$

to estimate the time-varying T_2_*, and obtain the T_2_* corrected signal $S_{0}(t)$.

At present, while T_2_* correction is recommended for consideration if available on the scanner, there is insufficient evidence to deem it essential, particularly since the short TE used in dual-sequence AIF acquisitions already largely suppresses T_2_* effects.

#### 3.4.5 Baseline correction

Any signal in the LV or myocardium prior to contrast arrival, due to PD and coil sensitivity, is typically referred to as baseline or pre-contrast signal (or baseline contrast agent concentration after SI-to-contrast-agent-concentration conversion) and can impact quantification if unaccounted for. For example, a positive baseline signal in the LV can lead to an underestimation of MBF. Therefore, the baseline signal must be nulled by subtracting it from all time frames. The baseline value can be obtained either from the first time-point or from the average of multiple time-points prior to contrast arrival to improve the robustness in the case of low SNR. It is good practice to baseline-correct even after conversion to contrast agent concentration to remove the contribution of any remnant of contrast from previous perfusion scans [132]. This is particularly relevant when a rest acquisition is performed after the stress acquisition and there is residual contrast agent.

Based on current evidence, it is recommended to apply baseline correction to the SI-time curves, or to the contrast agent concentration curves after SI-to-contrast-agent-concentration conversion.

#### 3.4.6 Spatial and temporal filtering

The ability of first-pass perfusion sequences to acquire multi-slice images in real-time within each heartbeat has the drawback of potentially a low SNR, which may impact the precision of quantitative perfusion measurements. While filtering can enhance perfusion quantification at various stages, it must be applied with caution. One area where filtering can assist perfusion quantification is in the automatic detection of the tracer arrival time in the blood pool or myocardium (as described below), particularly when excessive noise in baseline dynamics may lead to detection errors. Previous studies have used spatial (2D or 3D) and temporal filtering methods, including low-pass filtering [133], Gaussian and median filtering, to remove high-frequency components from the signals and improve SNR. However, in perfusion quantification pipelines that employ pixel-wise fitting, temporal filtering has become computationally expensive and is often omitted, as the fitting process itself is relatively insensitive to noise. Furthermore, temporal filtering applied to the AIF images can reduce the peak signal and affect the accuracy of perfusion quantification. On the other hand, spatial filtering of the myocardial images may substantially improve MBF precision by leveraging information from neighboring pixels to correct outliers. Nevertheless, care should be taken to avoid introducing significant partial volume effects at the endocardial and epicardial wall borders that could adversely affect subsequent processing steps. It is thus recommended that spatial filtering is limited to the high-resolution myocardial slices, including PD-weighted images, and should be minimized or avoided if the SNR is sufficient for quantification. Temporal filtering is not recommended.

An important factor when using non-Cartesian, low-rank, compressed sensing, or deep-learning reconstructions is to ensure that the temporal profile of the first-pass signal remains substantially unaltered, as distortions might affect the resulting quantitative measurements.

Commercially available and other DL denoising/sharpening algorithms can further be employed for denoising of perfusion images and improved SNR, however, it is important to make sure that these algorithms do not distort the signal intensities or temporal fidelity of the data.

#### 3.4.7 LV detection and AIF sampling

The AIF curve is essential for perfusion quantification, acting as the input reference concentration of the contrast agent in the LV before it distributes to the myocardium. This curve reflects the principle of indicator dilution, where the contrast agent concentration in the LV is used to model its distribution throughout the myocardium, allowing for accurate measurement of MBF [127]. In the dual-bolus technique, the AIF is sampled within the LV blood pool from one of the short-axis slices, typically the basal slice. In the dual-sequence technique, it is sampled from the low-resolution slice placed at the basal level or in the proximal aorta. A variety of methods for detecting the LV cavity and sampling the AIF have been proposed in the literature. For example, standard deviation maps of the SI curves can be used to identify contrast-enhancing regions, which are then analyzed based on their temporal characteristics to identify the LV [134]. Additional processing steps ensure that papillary muscles and pixels at the endocardial wall borders, which may be impacted by partial volume effects, are excluded. While conventional image processing methods are valuable and utilized in automated pipelines, they may have a suboptimal performance in cases of poor image quality, low SNR, or in the presence of artifacts. Recent approaches using DL techniques have shown promise in more accurately detecting the LV and sampling the AIF [65], [66].

Although current evidence is insufficient to recommend any specific approaches for LV detection and AIF sampling, machine learning and particularly DL is likely to enable faster and more robust automated LV detection. Regardless of the approach used, if the AIF is sampled in the LV, it must be obtained from a region that excludes the papillary muscles and any areas affected by partial volume effects from pixels at the endocardium.

#### 3.4.8 Myocardial segmentation

Tracing the endocardial and epicardial wall borders is not essential for quantitative analysis but can help to reduce the processing time by focusing MBF quantification on the myocardium while excluding surrounding tissues. When coupled with the detection of the right-ventricular (RV) insertion points, myocardial segmentation can also allow the generation of segmental bullseye plots according to the American Heart Association (AHA) model [135].

Automated methods for myocardial segmentation have recently been developed [65], [136], [137]. These are based on either training a U-Net DL model [138] or using a conventional computer vision approach. They differ, however, in terms of the input data given to the model. Segmentation can be performed on the MBF map [137], a single time frame of the image series at LV peak enhancement [139], the full temporal sequence of images [136], or a combination of the MBF map and the full perfusion image series [140]. Each approach has its advantages and disadvantages. For example, the segmentation of the MBF map is likely to be robust as the myocardium can be depicted within a physiologically meaningful MBF range. However, MBF quantification must be performed first over a larger image region, making the segmentation unable to inform or accelerate the quantification. In contrast, segmentation of a single time frame relies on adequate motion correction to be effective.

Current evidence is insufficient to recommend any specific approach for myocardial segmentation, but machine learning will likely improve this process. Segmented endocardial and epicardial contours should be devoid of contamination from blood pool or extracardiac tissues. Special attention should be given to the apical slice, where the reduced thickness of the myocardium and increased partial volume effects can complicate an accurate segmentation.

### 3.5 Quantification methods

#### 3.5.1 Perfusion modeling

For perfusion quantification, the tissue is modelled as a system with inlets and outlets [141]. The models are built on the tracer-kinetic theory of linear time-invariant systems [142]. That is, the transit time (i.e., the time elapsed between entering and leaving the system), does not depend on the time of the contrast injection or the injected concentration. The system is governed by the law of conservation of mass, which states that no tracer (the contrast agent is referred to as a tracer and an indicator interchangeably) is created or destroyed in the system and, accordingly, the rate of change of concentration in the tissue is the difference between the influx and outflux through the inlets and outlets of the system:

(3) $$v\frac{d\;C(t)}{\mathrm{dt}}=\sum_{i}F_{i}C_{i}\left(t\right)-\sum_{o}F_{o}C_{o}\left(t\right)\;$$

where v is volume of distribution (the volume of the system that contains the tracer), C(t) is the total concentration of contrast in the system and, $F_{i}$, $\;F_{o}$ and $C_{i}\left(t\right)$, $C_{0}\left(t\right)$ are the flows through and concentrations at the inlets i and outlets o of the system. The system itself can be made up of several interacting compartments with the inlets of a compartment possibly being the outlets of another compartment. Eq. 3 can be applied to each compartment to yield a system of coupled ordinary differential equations. It is assumed that for a myocardial region of interest the arterial inputs are functionally equivalent, meaning there is no spatial variation of the AIF along the boundaries of the tissue region [143]. The input to the system is the AIF ($C_{\mathrm{AIF}})$ and the output is the myocardial concentration curve ($C_{\mathrm{myo}})$. A vast array of models can then be derived by making different assumptions on the number of interacting compartments in the system and their properties.

There has been no definitive comparison between the possible models and there is currently no clear consensus on the ‘best’ model. It has been suggested that the two-compartment exchange model (2CXM) may be suitable [144]. For this model, the tracer is assumed to be contained in the blood plasma and the extravascular extracellular space, and each of these spaces is an individual, well-mixed compartment. The influx and outflux of the system are assumed to be only through the plasma compartment, the extravascular extracellular compartment only exchanges with the plasma compartment, and this exchange is equal in both directions.

**Fig. 4:**
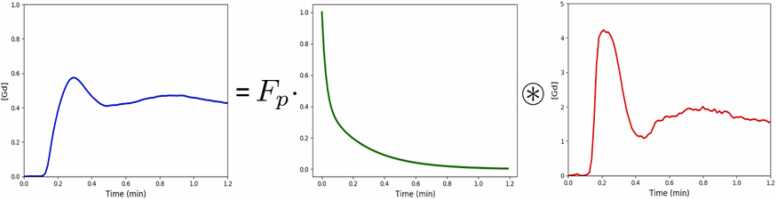
The myocardial tissue curve (blue) is shown as the result of a convolution of the AIF (red) with a residue curve (green). *AIF* arterial input function.

In blood, the plasma concentration of contrast agent is derived from the measured or estimated T_1_ in blood and the hematocrit. Typically, despite different assumed models, an analytic solution for $C_{\mathrm{myo}}\left(t\right)$ in terms of a convolution with a residue function can be obtained using the Laplace transform and is shown in Fig. 4, under the assumption of zero initial concentration such that:

(4) $$C_{\mathrm{myo}}\left(t\right)=F_{p}\cdot R\left(t\right)*C_{\mathrm{aif}}(t)$$

where *F*_*p*_ is plasma flow, with the residue function $R\left(t\right)$ changing depending on the model assumptions. It is only for distributed-parameter models (in which concentrations depend on some spatial coordinate as well as time, e.g. to model a contrast concentration gradient from arterial to venous side) that an analytic solution is not available in the time domain.

Given the assumption of linear time-invariance, it is natural that the solution is in the form of a convolution and, indeed, Eq. 4 can be reached without the assumption of a compartmental model based on the central volume principle [145]. Direct deconvolution of $C_{\mathrm{myo}}$ with $C_{\mathrm{aif}}$then gives the impulse response function of the system $I\left(t\right)=F_{p}R\left(t\right)$, which is a monotonically decreasing curve. Since the interpretation of the residue function is the fraction of tracer particles that are still present at time t, $R\left(0\right)=1$ and thus MBF can be found as $F_{p}=I(0)$. Such an approach is described as model-independent. Mathematically these methods are ill-posed, meaning that the solution is extremely sensitive to changes in the data. For this reason, the solution has to be constrained, for example, by applying Tikhonov regularization [48], [146]. It can also be constrained by representing the impulse response function as a parameterized curve such as the Fermi function [31]. In myocardial perfusion modeling, the Fermi function was chosen as it was found to provide a relatively simple approximation to the initial rapid vascular transit of contrast through tissue after an impulse input [147]. The general formula for the Fermi function is [144]:

(5) $$I\left(t\right)=\frac{A}{e^{\frac{t-\mu }{k}}+1}$$

The Fermi function can encapsulate varying response functions with different tracer transit times, governed by its magnitude A, the initial plateau width or temporal delayμ, and the contrast residence time or decay rate k. Recent implementations have added a fourth offset parameter to account for CA leaking in the interstitial space and improve fitting accuracy [46], [148].

An important consideration in perfusion modeling is the sampling rate of the acquired data. Since the measurements are synchronized to the patient’s cardiac cycle using ECG triggering, the sampling rate may not be uniform. A recent study demonstrated that abnormally high or low sampling rates lead to errors in deconvolution analysis and consequently to potentially significant errors in MBF quantification [149].

#### 3.5.2 Parameter quantification

Prior to solving the convolution Eq. 4 and estimating MBF, the AIF is first examined to remove baseline time points before contrast agent arrival in the blood pool (start of the AIF upslope). Perfusion models describing a single vascular compartment can accurately fit only on the first-pass portion of the curves and require removal of contrast agent recirculation, whereas multi-compartment models can be fitted to the whole dataset. Once appropriate timing and the duration of first pass are determined, individual tissue curves can also be processed accordingly. Automated algorithms for detecting the tracer arrival time in the AIF include the triangle method [150] and fitting a line to the upslope [134], amongst others. Then, model parameters can be estimated using the observed myocardial and arterial concentration curves by employing nonlinear least-squares fitting [151], with the most popular choice of fitting algorithm being the Levenberg–Marquardt approach [152], [153]. Accordingly, the sum of squared errors between the analytic model for $C_{\mathrm{myo}}$ and the observed myocardial tissue concentration curves is minimized with respect to the model parameters.

The ability to accurately estimate model parameters depends on the quality of the data. Quality can be limited by SNR, the acquisition time, the temporal resolution, and artifacts such as motion [151]. The reliability of the estimated parameters can also be improved by reducing the complexity of the model used [154]. As previously mentioned, many choices of models for myocardial perfusion quantification are possible, and include the Kety-Tofts model [155] and the Fermi function [156]. Patlak plot is another alternative, which is derived from the Kety-Tofts model, simplifying the analytical procedures [105], [157]. These models have fewer parameters to estimate than the 2CXM, making the fitting problem more stable. However, the Kety-Tofts model does not solve directly for$\;F_{p}$ and the parameters of the Fermi function do not assume a physiological meaning.

Another approach to deal with the low SNR of tissue curves at the pixel level and improve the robustness of quantification is to iterate the cropping and fitting process for multiple time points after contrast agent arrival in the blood pool and select the arrival time in the myocardium providing the smallest fitting residuals [66], [112], [142], [158]. Fitting a multi-compartment exchange model in two steps, in a coarse-to-fine approach, has been proposed to try to avoid local minima [66]. In the first step the best set of parameters over a pre-defined range of parameters is searched for and this estimate is then used as an initialization for a Nelder–Mead simplex optimization. A different approach is to increase the amount of information available in the data using prior knowledge to reduce uncertainty. For example, spatial Tikhonov regularization [146] or spatial prior knowledge can be incorporated in a Bayesian framework [139]. There has also been a limited amount of work using DL to directly predict the parameters [159], [160]. Alternatively, parameter estimates can be improved by fitting to a concentration curve averaged over a whole segment of myocardium. This sacrifices resolution in order to increase SNR, but it is now well-known that high-resolution maps are needed for detecting subtle subendocardial ischemia [161], [162]. Therefore, segment-wise quantification is not recommended in this consensus statement.

To date, only a small number of studies [157], [163] have compared different quantification models including Fermi-function deconvolution, model-independent approaches and tracer-kinetic modelling (including distributed-parameter models). In general, models which have fewer parameters to fit—such as the Fermi model—tend to be more robust, but the most accurate method for quantification has not been established. At present, there is insufficient evidence to recommend a specific quantification model.

### 3.6 Interpretation

First-pass perfusion images should be reviewed visually for diagnostic purposes in accordance with current guidelines [14]. The presence of myocardial scar should be determined from late gadolinium enhancement (LGE) images rather than rest perfusion images or quantitative rest perfusion maps. There is no current evidence on how to integrate visual and quantitative myocardial perfusion analysis. Perfusion images and maps should be reviewed side-by-side or superimposed with the ability to quickly change from quantitative maps to perfusion images. Artifacts, perfusion defects and other apparent findings on perfusion images should be considered when interpreting quantitative perfusion results.

Pixel-wise perfusion maps should be related to coronary perfusion territories using the AHA 16 segment model [135] as guidance, even though the correspondence between the 16-segment model and coronary artery territories is more complicated than initially described. More recent interpretations conclude that many sectors can be attributed to more than one basic coronary artery territory [164]. Cut-off values for abnormal MBF vary across implementations and need to be standardized. Exact thresholds for a normal MPR are not universally accepted currently but are generally on the order of an MPR of 2. Some authors have advocated the use of the use of sex- and age-specific reference ranges for diagnostic use [2]. The diagnostic and prognostic utility of rest MBF remains a topic of debate. However, MPR assessment, which is necessary for assessing functional microvascular disease, requires rest perfusion imaging [14], [38]. In case of structural microvascular disease, combined stress and rest perfusion imaging and LGE imaging may be required to provide the correct diagnosis [59]. A detailed discussion of the complex interactions between how oxygen demand and perfusion are regulated are beyond the scope of this technical paper.

In analogy to current practice during visual assessment, we recommend quantifying the size of perfusion defects in terms of percent of myocardium affected rather than number of segments affected. Note that larger perfusion defects are prognostically significant particularly if affecting 2 or more segments [69], [165], corresponding to >12% (2 segments divided by 16 segments * 100%) of the myocardium. Online Supplement 1 demonstrates typical patterns observed on quantitative perfusion maps in comparison with the corresponding first pass series.

### 3.7 Quality control

**Fig. 5:**
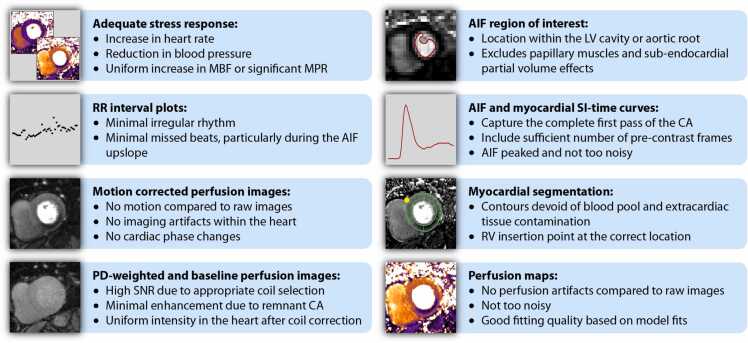
Main quality control steps in myocardial perfusion quantification.

It is recommended to follow a systematic approach to assess the quality of the data acquired and of the perfusion maps generated, as summarized in Fig. 5. While each step in the image processing and data analysis can be (and should be) automated, it is important to remember that no automated algorithm can perform optimally in 100% of cases. This section describes key steps that should be checked to ensure high-quality results. Ideally the following elements should be reviewed in all cases.

#### 3.7.1 Clinical response

Symptoms recorded during the data acquisition should be reviewed to ensure that there was adequate clinical response during stress imaging (heart rate increase, patient symptoms, changes in blood pressure). The time between stress and rest image acquisition should be considered. Plots of the RR interval should be reviewed for irregular rhythm or missed beats, which could corrupt quantification if they occur during the upslope of the AIF or the myocardial SI-time curve. In the case that issues arise with the acquisition of the stress scan, the acquisition could be repeated, replacing the rest scan. A sufficient interval should be allowed between the first and the second acquisition.

#### 3.7.2 Image quality

The unprocessed myocardial and AIF images should be visually checked for artifacts. Verify that the AIF region of interest is properly localized in the LV cavity or aortic root and excludes papillary muscles and endocardial partial volume effects. Motion-corrected myocardial and AIF images, including PD images, should be displayed as cine loops to determine success of motion correction. Including the PD images in the cine loops might cause a flash-like appearance which might impact visual interpretation of perfusion studies. A cine loop without PD images can be considered as an additional output series, provided that motion-corrected myocardial and AIF images, including PD images, are also exported. Pre-contrast frames (including PD-weighted and baseline perfusion images) should also be checked for adequate SNR and signal uniformity after coil sensitivity correction. An enhanced blood pool in baseline perfusion images is evidence of remnant contrast agent from a previous perfusion scan and is often indicative of an insufficient delay between acquisitions or low cardiac output of a patient.

#### 3.7.3 Curve quality

Whenever possible, plots of the raw and motion-corrected myocardial and AIF curves should be reviewed to determine any registration issues and ensure a sufficient number of pre-contrast frames are available for baseline correction. The contrast arrival times for the AIF and myocardial curves should be verified and the start points of the AIF and myocardial tissue response should be compared. The AIF should be a peaked curve without apparent saturation and capture the complete first pass of the contrast agent. A low SNR AIF may indicate issues with injection, bolus preparation errors, incorrect placement of the AIF region of interest, or problematic sequence parameters, such as short TS or incorrect coil selection. In the case of issues with the acquisition of the stress scan, the acquisition could be repeated, replacing the rest scan. Finally, the quality of the fitting achieved by the quantification model can be checked to verify the quality of the results.

#### 3.7.4 Map quality

The quality of the perfusion maps should be assessed. Quantitative perfusion maps that show a uniform increase in MBF from rest to stress or a significant MPR are excellent quality assurance metrics to confirm both an adequate vasodilator response and a true negative stress perfusion test. Particular attention should be paid in cases where the maps look different in shape or wall thickness than the raw images, which can be indicative of poor motion correction or cardiac phase changes during the acquisition.

#### 3.7.5 Segmental analysis

When performing myocardial segmentation, positioning of the endocardial and epicardial contours should be reviewed to ensure that there is no blood pool or extracardiac tissue contamination, in particular in the most basal slice where outflow tract inclusion is common, and where the LV myocardium is thin either due to pathology or at the apex. Ensure that the right-ventricular insertion points for AHA segmentation are in the correct location for accurate orientation of the myocardial segments. Review of the perfusion maps to identify individual segments with image artifacts affecting the quality of the quantification is also recommended. These segments should be excluded from clinical interpretation. Online Supplement 2 outlines the critical aspects of quality control and the primary sources of error that can impact the accuracy and precision of quantitative perfusion mapping.

## 4 Gaps in knowledge

Although quantitative myocardial perfusion analysis is rapidly nearing clinical application, several knowledge gaps remain that would benefit from future dedicated clinical studies. These are outlined below:

1. The permissible degree of spatial filtering and the resulting minimum level of SNR required for quantification are not yet known.
2. The optimal fitting parameters to ensure the accuracy and repeatability of quantitative values and the comparison between algorithms would benefit from future prospective studies.
3. The effect of missed heart beats has the potential to corrupt quantification. The robustness of different quantification algorithms to missing data points should be tested and reported.
4. Optimal MBF and MPR cut-off values would need to be standardised across different quantification approaches, considering clinically relevant subgroups.
5. The percentage of ischemic myocardium that indicates the need for revascularisation should be validated in prospective outcome studies.

## 5 Conclusion and future directions

This SCMR expert consensus document provides recommendations for the acquisition and analysis of quantitative myocardial perfusion CMR based on current evidence and expert opinion. This document is to provide guidance to the community and industry on how to approach quantitative myocardial perfusion CMR development and facilitate standardization of methodology across an increasing number of platforms.

It is explicitly acknowledged that several fundamental questions remain open and require future research. These include the optimal model for quantification over the entire range of MBF values encountered in clinical practice, the relative benefits of spatial coverage, spatial resolution, and temporal resolution versus SNR sacrifices associated with increasing acceleration. Also, the impact of CS and DL-based approaches on spatiotemporal fidelity of the data remains to be studied.

While Cartesian acquisition has been the mainstay of clinical imaging, spiral and radial techniques may be more efficient and have reduced sensitivity to motion, warranting further investigation. Emerging methods allow SMS or 3D acquisition, which may herald advantages by reducing through-plane motion inconsistency in distinct phases of the cardiac cycle.

Image quality and consequently the accuracy of perfusion quantification are significantly affected by various sources of error inherent in MRI acquisitions. Specifically, magnetic field inhomogeneities, imperfect pulses, chemical shift, and partial volume effects will influence the images. Many of these effects are currently not or only incompletely accounted for.

The document does not cover the indications or clinical use of quantitative myocardial perfusion CMR, which will be the subject of a future SCMR consensus document.

## Author contributions

**Amedeo Chiribiri:** Conceptualization, writing – original draft, writing – review & editing. **Andrew E. Arai:** Conceptualization, writing – original draft, writing – review & editing. **Edward DiBella:** Conceptualization, writing – original draft, writing – review & editing. **Li-Yueh Hsu:** Conceptualization, writing – original draft, writing – review & editing. **Masaki Ishida:** Conceptualization, writing – original draft, writing – review & editing. **Michael Jerosch-Herold:** Conceptualization, writing – original draft, writing – review & editing. **Sebastian Kozerke:** Conceptualization, writing – original draft, writing – review & editing. **Xenios Milidonis:** Conceptualization, writing – original draft, writing – review & editing. **Reza Nezafat:** Conceptualization, writing – original draft, writing – review & editing. **Sven Plein:** Conceptualization, writing – original draft, writing – review & editing. **Cian M. Scannell:** Conceptualization, writing – original draft, writing – review & editing. **Michael Salerno:** Conceptualization, writing – original draft, writing – review & editing.

## Declaration of competing interests

The authors declare the following financial interests/personal relationships which may be considered as potential competing interests. Andrew Ernest Arai reports a relationship with Circle Cardiovascular Imaging Inc that includes speaking and lecture fees and travel reimbursement. Sebastian Kozerke reports a relationship with Philips Healthcare Netherlands that includes funding grants. Reza Nezafat reports a relationship with Siemens Healthineers AG that includes non-financial support. Michael Salerno reports a relationship with Siemens Healthineers AG that includes non-financial support. Michael Salerno reports a relationship with GE Healthcare that includes non-financial support. Amedeo Chiribiri reports a relationship with Siemens Healthineers AG that includes non-financial support. Amedeo Chiribiri reports a relationship with Philips Healthcare United Kingdom that includes funding grants. Amedeo Chiribiri reports a relationship with GE Healthcare that includes consulting or advisory. Amedeo Chiribiri reports a relationship with Circle Cardiovascular Imaging Inc that includes non-financial support. Andrew Ernest Arai reports a relationship with Bayer AG that includes: speaking and lecture fees and travel reimbursement. Li-Yueh Hsu has patent Software for myocardial perfusion quantification licensed to No IP rights or licences are currently active. Cian Scannell has patent #Method and System for Estimating Arterial Input Function / US20230253110A1 pending to King’s College London. Amedeo Chiribiri has patent #Method and System for Estimating Arterial Input Function / US20230253110A1 pending to King’s College London. Dr Reza Nezafat - Editorial board of JCMR, MRM and JMRI. Executive Editor for JMRI Research Funding from NIH. Given his/her/their role as, had no involvement in the peer review of this article and had no access to information regarding its peer review. Full responsibility for the editorial process for this article was delegated to another journal editor. If there are other authors, they declare that they have no known competing financial interests or personal relationships that could have appeared to influence the work reported in this paper.
