# Supplementary material for: Society for cardiovascular magnetic resonance expert consensus statement on quantitative myocardial perfusion cardiovascular magnetic resonance imaging
Source: J Cardiovasc Magn Reson. 2025 Aug 8;27(2):101940. doi: 10.1016/j.jocmr.2025.101940 (PMC12766621; doi:10.1016/j.jocmr.2025.101940)
Supplement: Supplementary file 1 — Supplementary material [file mmc1.pptx]

## Slide 1
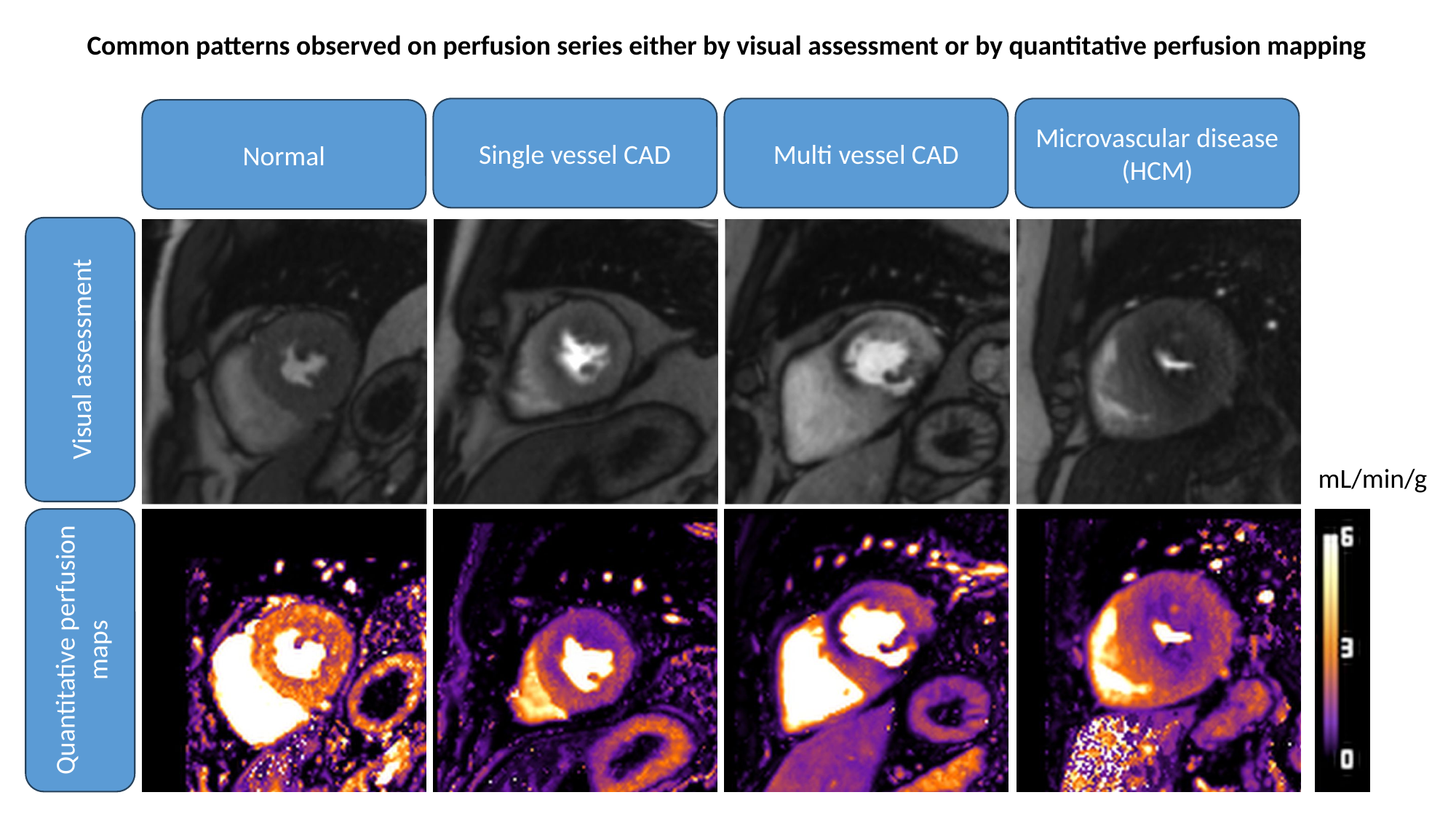

Common patterns observed on perfusion series either by visual assessment or by quantitative perfusion mapping
Single vessel CAD
Multi vessel CAD
Microvascular disease
(HCM)
Normal
Visual assessment
mL/min/g
Quantitative perfusion maps
