# Supplementary material for: Society for cardiovascular magnetic resonance expert consensus statement on quantitative myocardial perfusion cardiovascular magnetic resonance imaging
Source: J Cardiovasc Magn Reson. 2025 Aug 8;27(2):101940. doi: 10.1016/j.jocmr.2025.101940 (PMC12766621; doi:10.1016/j.jocmr.2025.101940)
Supplement: Supplementary file 3 — Supplementary material [file mmc3.pptx]

## Slide 1
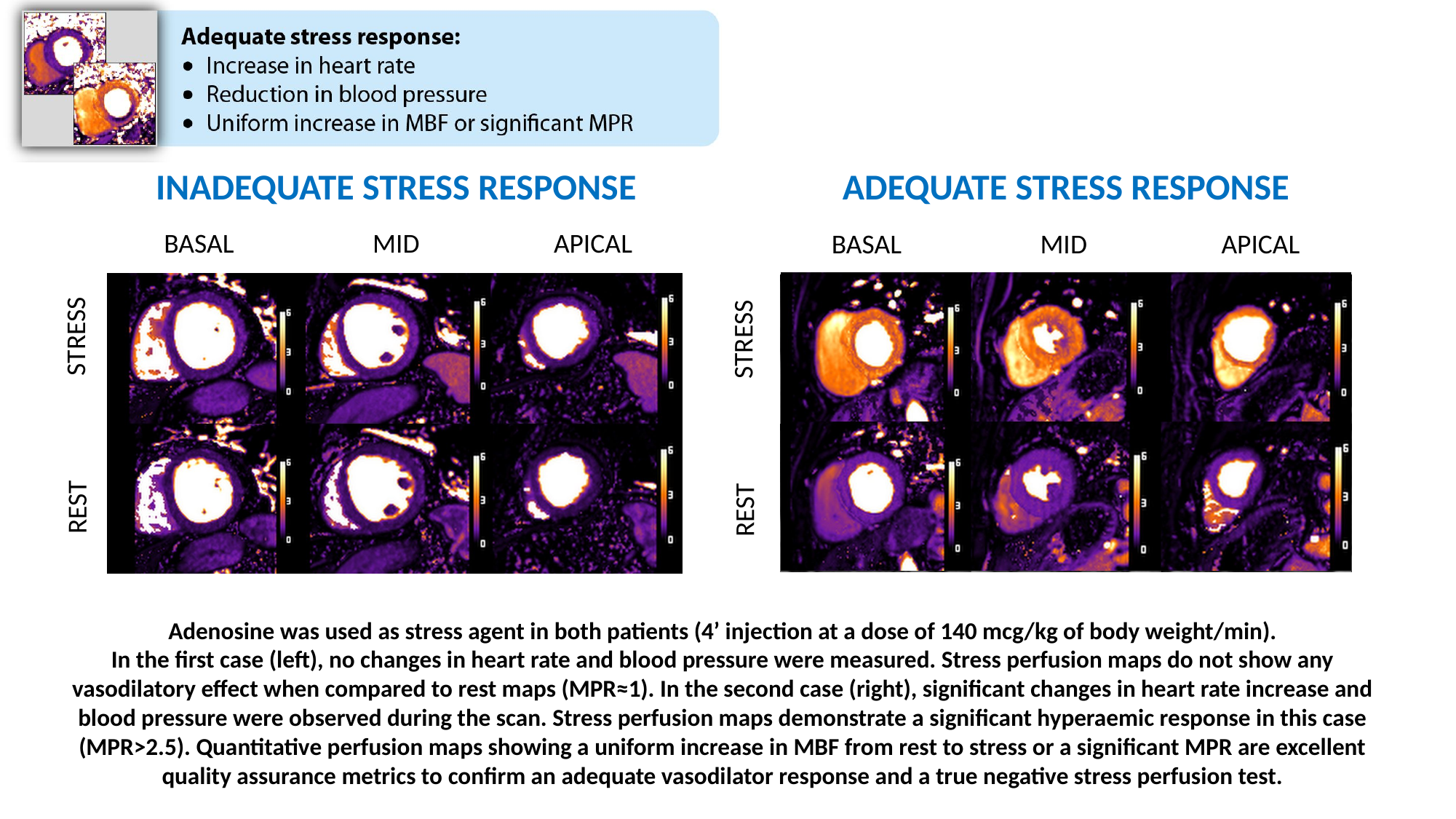

INADEQUATE STRESS RESPONSE
ADEQUATE STRESS RESPONSE
BASAL
MID
APICAL
STRESS
REST
BASAL
MID
APICAL
STRESS
REST
Adenosine was used as stress agent in both patients (4’ injection at a dose of 140 mcg/kg of body weight/min).
In the first case (left), no changes in heart rate and blood pressure were measured. Stress perfusion maps do not show any vasodilatory effect when compared to rest maps (MPR≈1). In the second case (right), significant changes in heart rate increase and blood pressure were observed during the scan. Stress perfusion maps demonstrate a significant hyperaemic response in this case (MPR>2.5). Quantitative perfusion maps showing a uniform increase in MBF from rest to stress or a significant MPR are excellent quality assurance metrics to confirm an adequate vasodilator response and a true negative stress perfusion test.

## Slide 2
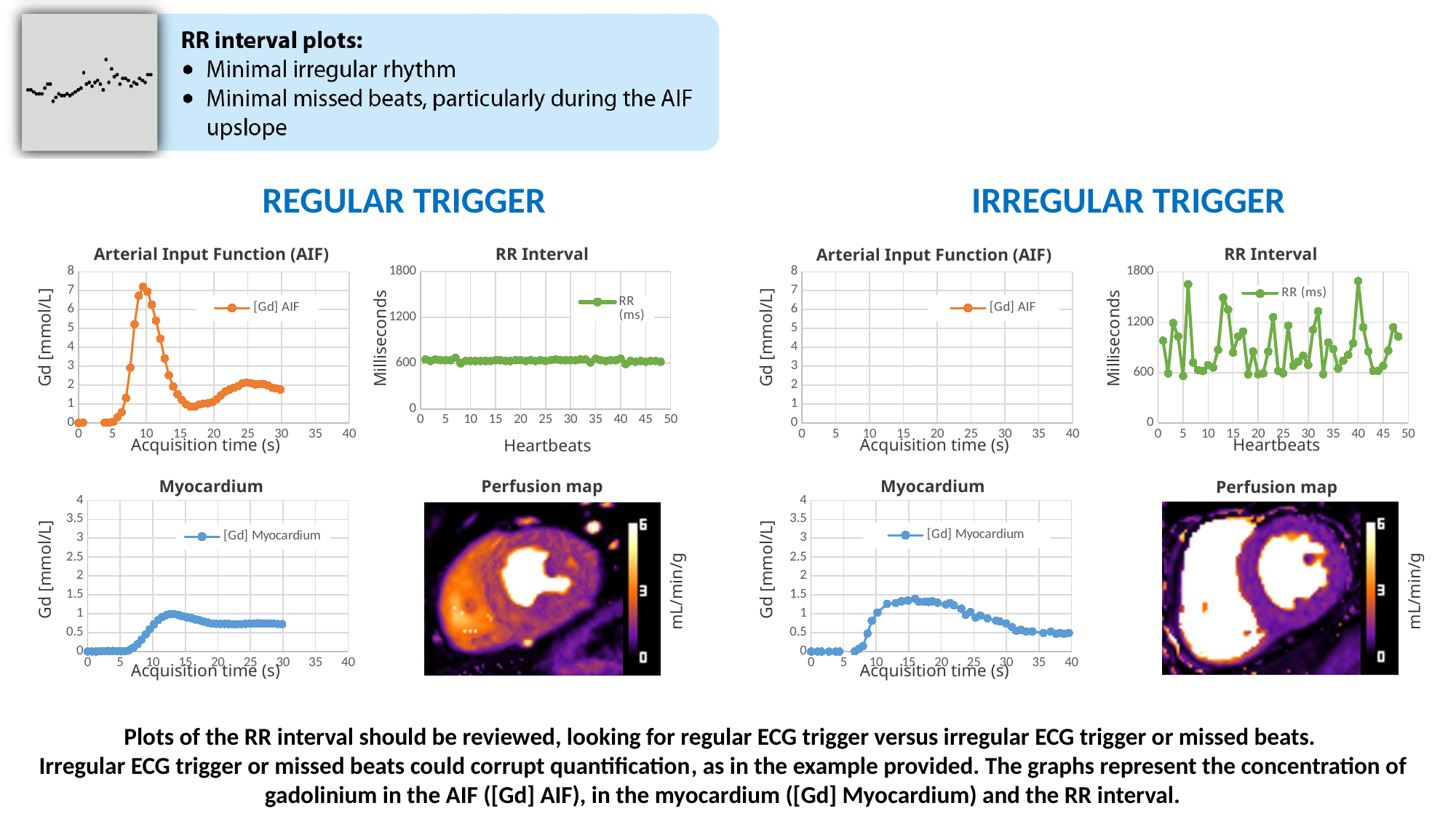

REGULAR TRIGGER
IRREGULAR TRIGGER
Arterial Input Function (AIF)
RR Interval
RR Interval
Arterial Input Function (AIF)
### Chart
| Category | RR (ms) |
|---|---|
### Chart
| Category | [Gd] AIF |
|---|---|
### Chart
| Category | [Gd] AIF |
|---|---|
### Chart
| Category | RR (ms) |
|---|---|Gd [mmol/L]
Gd [mmol/L]
Milliseconds
Milliseconds
Acquisition time (s)
Acquisition time (s)
Heartbeats
Heartbeats
Myocardium
Myocardium
Perfusion map
Perfusion map
### Chart
| Category | [Gd] Myocardium |
|---|---|
### Chart
| Category | [Gd] Myocardium |
|---|---|
Gd [mmol/L]
Gd [mmol/L]
mL/min/g
mL/min/g
Acquisition time (s)
Acquisition time (s)
Plots of the RR interval should be reviewed, looking for regular ECG trigger versus irregular ECG trigger or missed beats.
Irregular ECG trigger or missed beats could corrupt quantification, as in the example provided. The graphs represent the concentration of gadolinium in the AIF ([Gd] AIF), in the myocardium ([Gd] Myocardium) and the RR interval.

## Slide 3
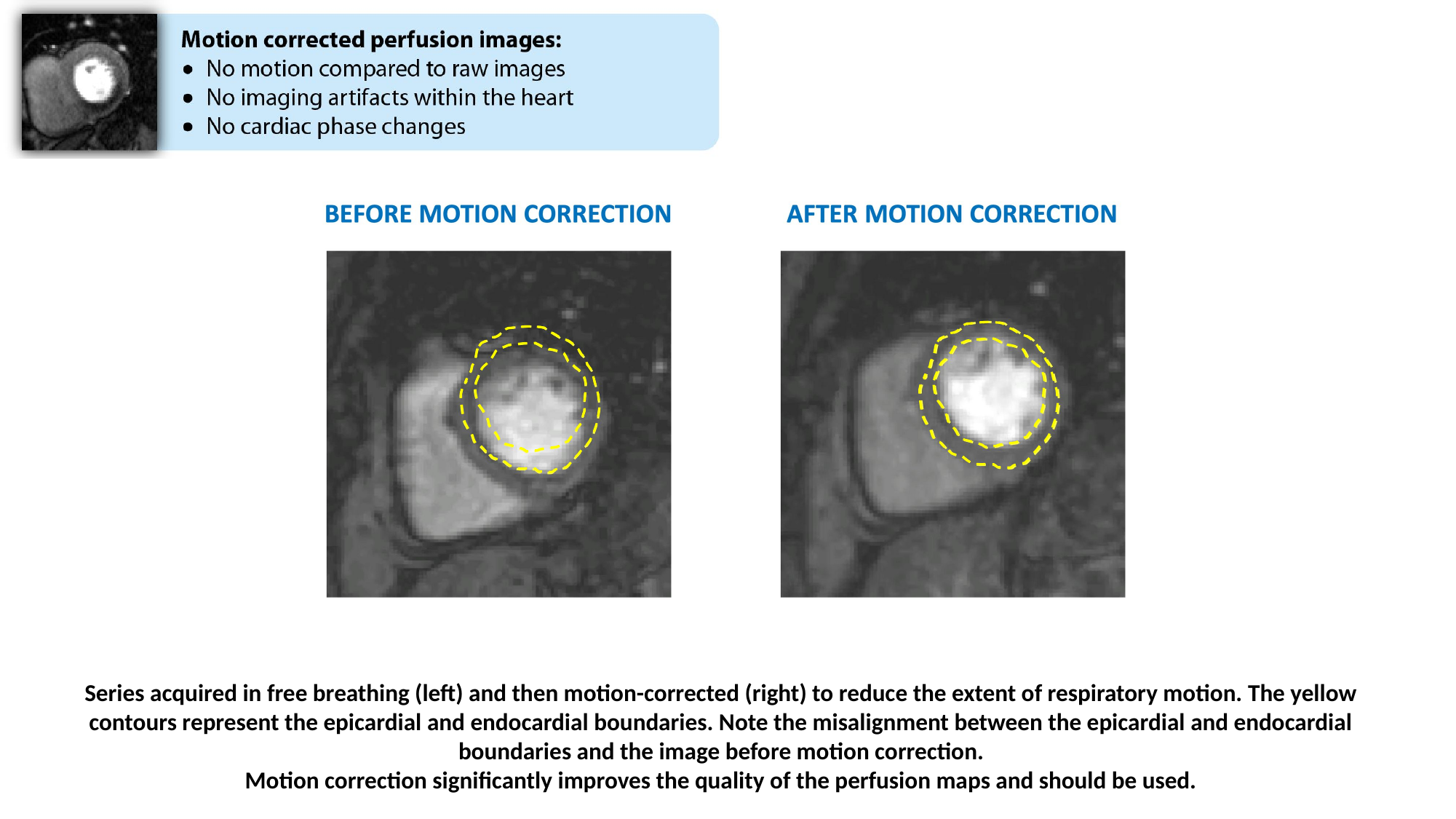

Series acquired in free breathing (left) and then motion-corrected (right) to reduce the extent of respiratory motion. The yellow contours represent the epicardial and endocardial boundaries. Note the misalignment between the epicardial and endocardial boundaries and the image before motion correction.
Motion correction significantly improves the quality of the perfusion maps and should be used.

## Slide 4
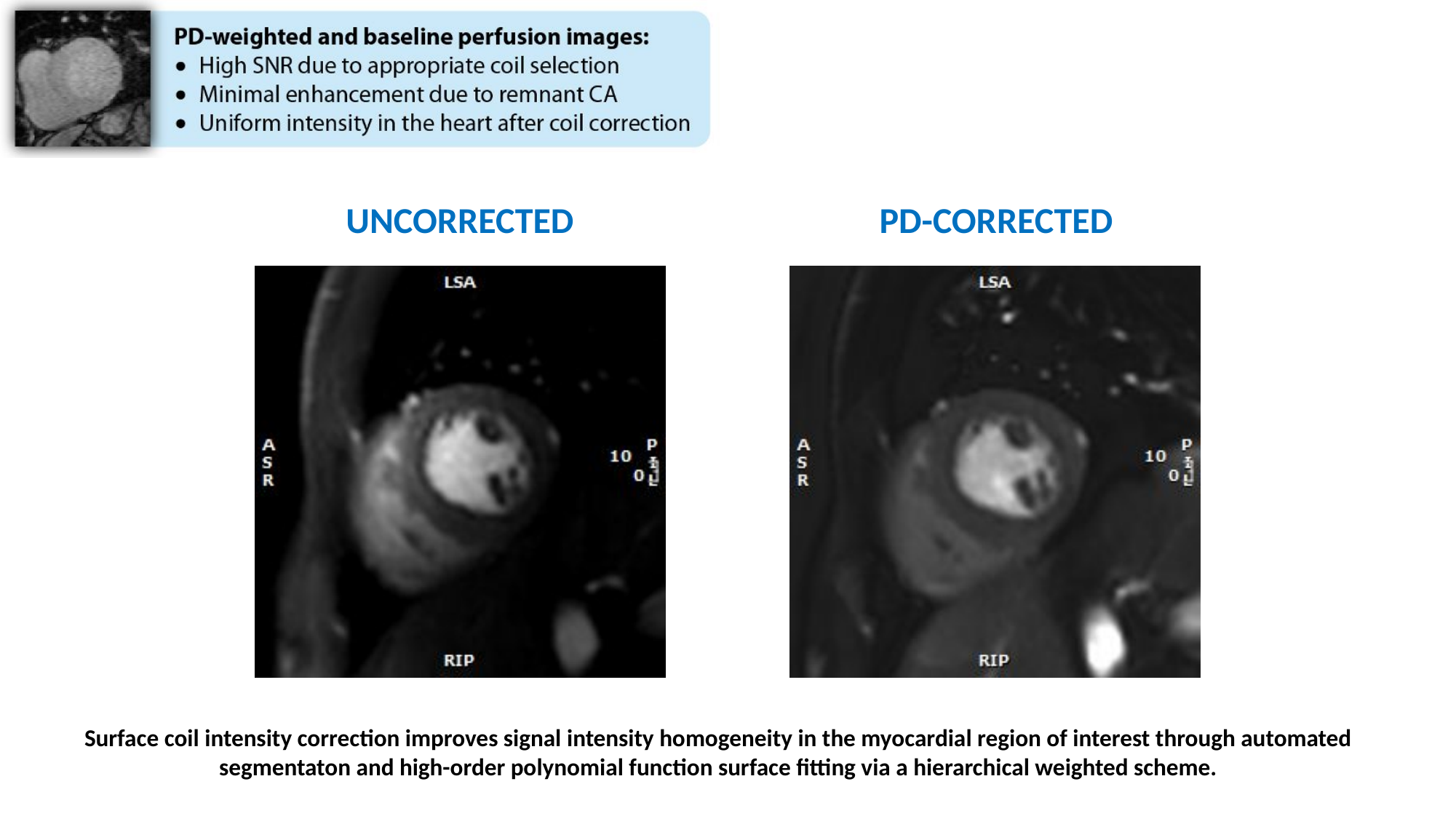

PD-CORRECTED
UNCORRECTED
Surface coil intensity correction improves signal intensity homogeneity in the myocardial region of interest through automated segmentaton and high-order polynomial function surface fitting via a hierarchical weighted scheme.

## Slide 5
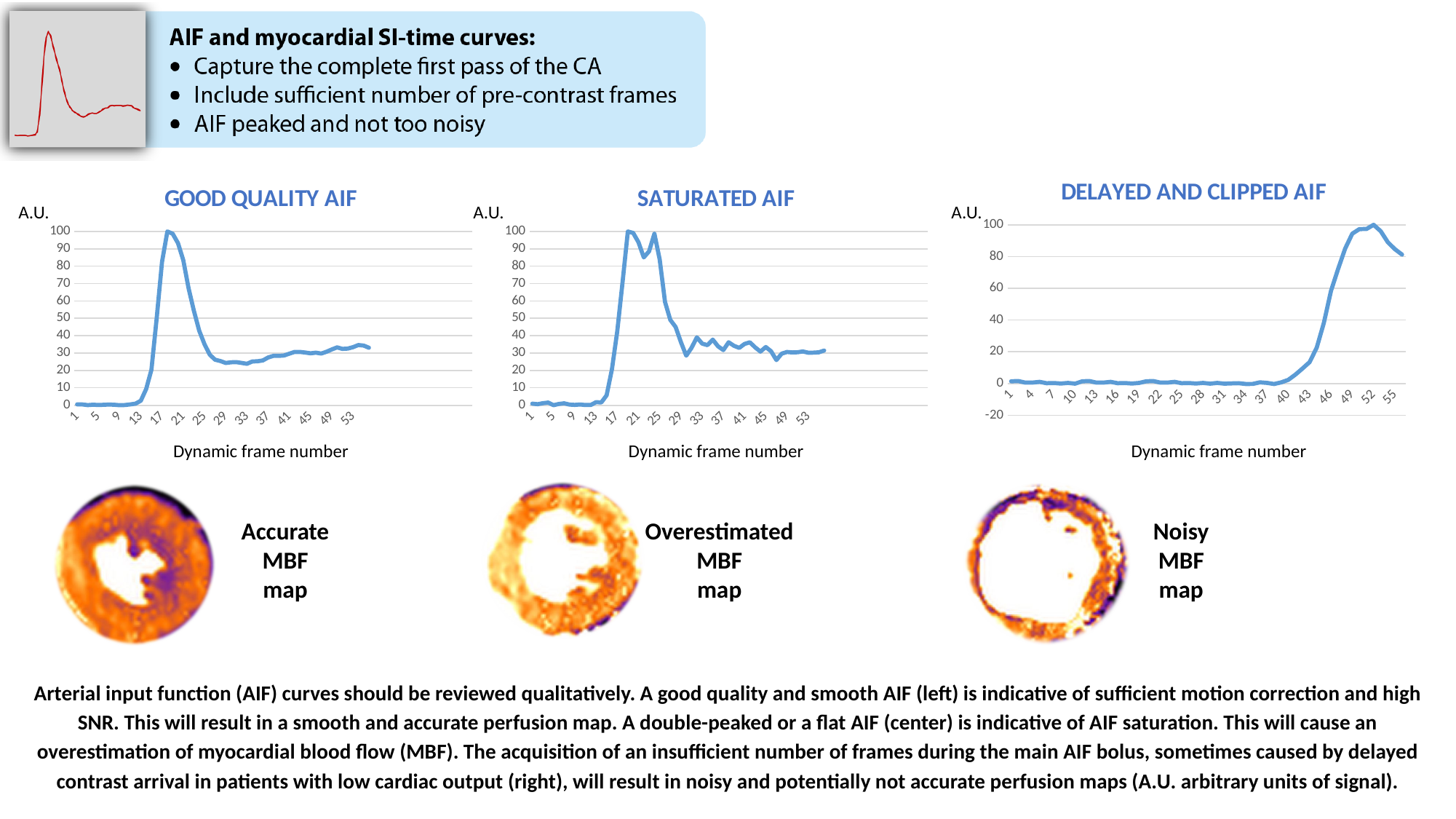

### Chart: DELAYED AND CLIPPED AIF
| Category | Normalised AIF (a.u.) |
|---|---|
### Chart: SATURATED AIF
| Category | Normalised AIF (a.u.) |
|---|---|
### Chart: GOOD QUALITY AIF
| Category | Normalised AIF (a.u.) |
|---|---|A.U.
A.U.
A.U.
Dynamic frame number
Dynamic frame number
Dynamic frame number
Overestimated
MBF
map
Noisy
MBF
map
Accurate
MBF
map
Arterial input function (AIF) curves should be reviewed qualitatively. A good quality and smooth AIF (left) is indicative of sufficient motion correction and high SNR. This will result in a smooth and accurate perfusion map. A double-peaked or a flat AIF (center) is indicative of AIF saturation. This will cause an overestimation of myocardial blood flow (MBF). The acquisition of an insufficient number of frames during the main AIF bolus, sometimes caused by delayed contrast arrival in patients with low cardiac output (right), will result in noisy and potentially not accurate perfusion maps (A.U. arbitrary units of signal).

## Slide 6
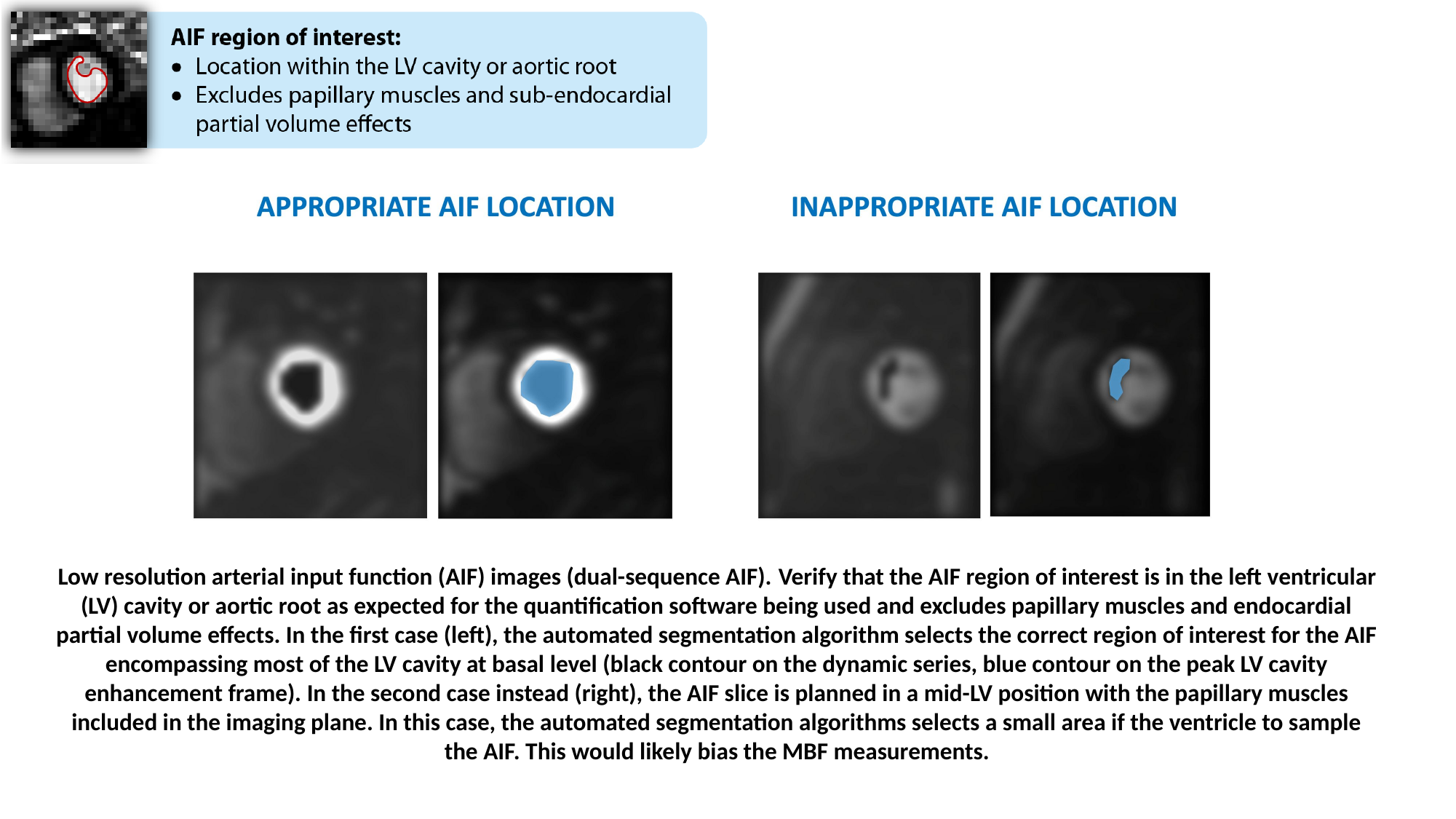

Low resolution arterial input function (AIF) images (dual-sequence AIF). Verify that the AIF region of interest is in the left ventricular (LV) cavity or aortic root as expected for the quantification software being used and excludes papillary muscles and endocardial partial volume effects. In the first case (left), the automated segmentation algorithm selects the correct region of interest for the AIF encompassing most of the LV cavity at basal level (black contour on the dynamic series, blue contour on the peak LV cavity enhancement frame). In the second case instead (right), the AIF slice is planned in a mid-LV position with the papillary muscles included in the imaging plane. In this case, the automated segmentation algorithms selects a small area if the ventricle to sample the AIF. This would likely bias the MBF measurements.

## Slide 7
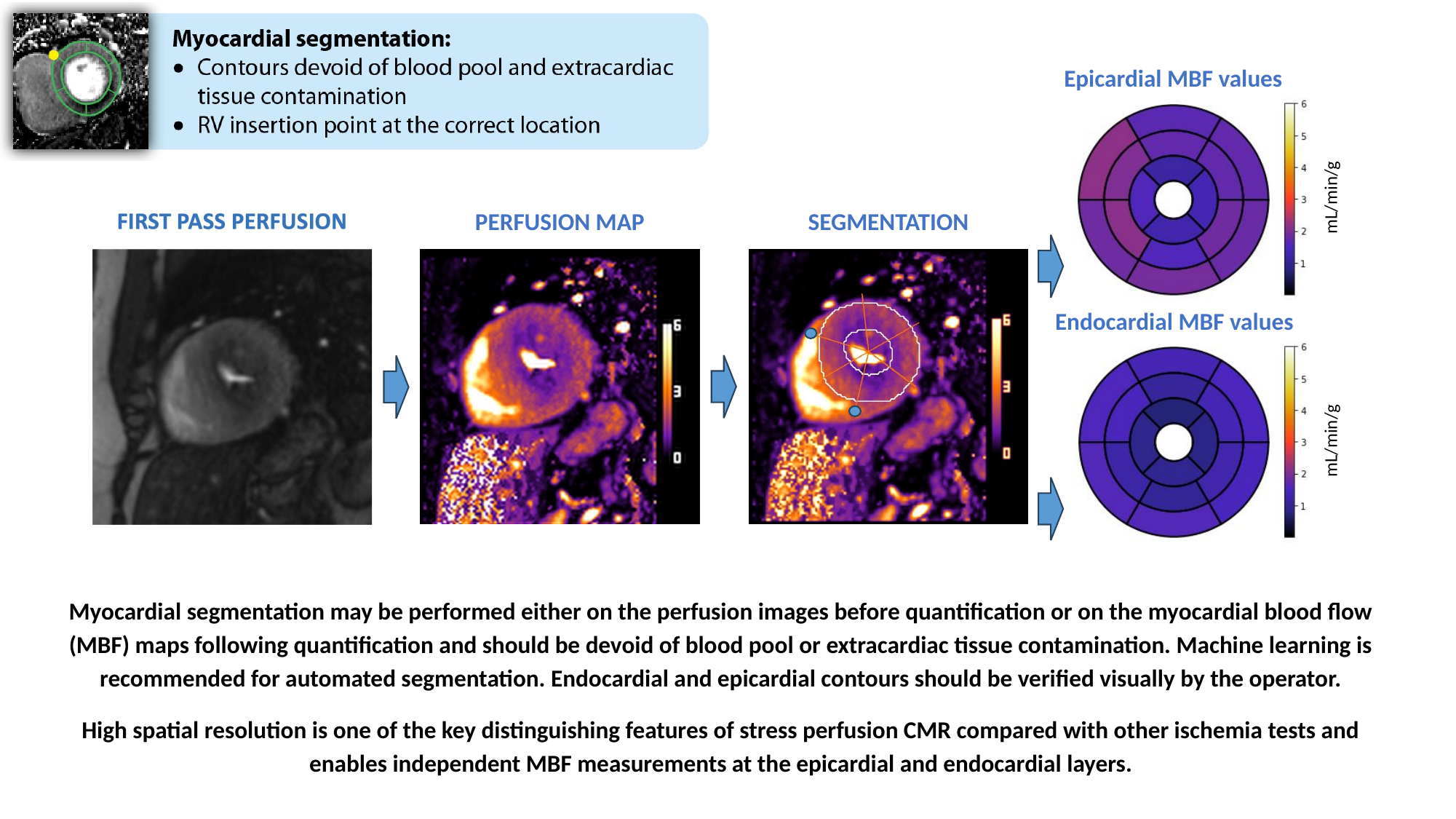

Epicardial MBF values
mL/min/g
PERFUSION MAP
SEGMENTATION
Endocardial MBF values
mL/min/g
Myocardial segmentation may be performed either on the perfusion images before quantification or on the myocardial blood flow (MBF) maps following quantification and should be devoid of blood pool or extracardiac tissue contamination. Machine learning is recommended for automated segmentation. Endocardial and epicardial contours should be verified visually by the operator.
High spatial resolution is one of the key distinguishing features of stress perfusion CMR compared with other ischemia tests and enables independent MBF measurements at the epicardial and endocardial layers.

## Slide 8
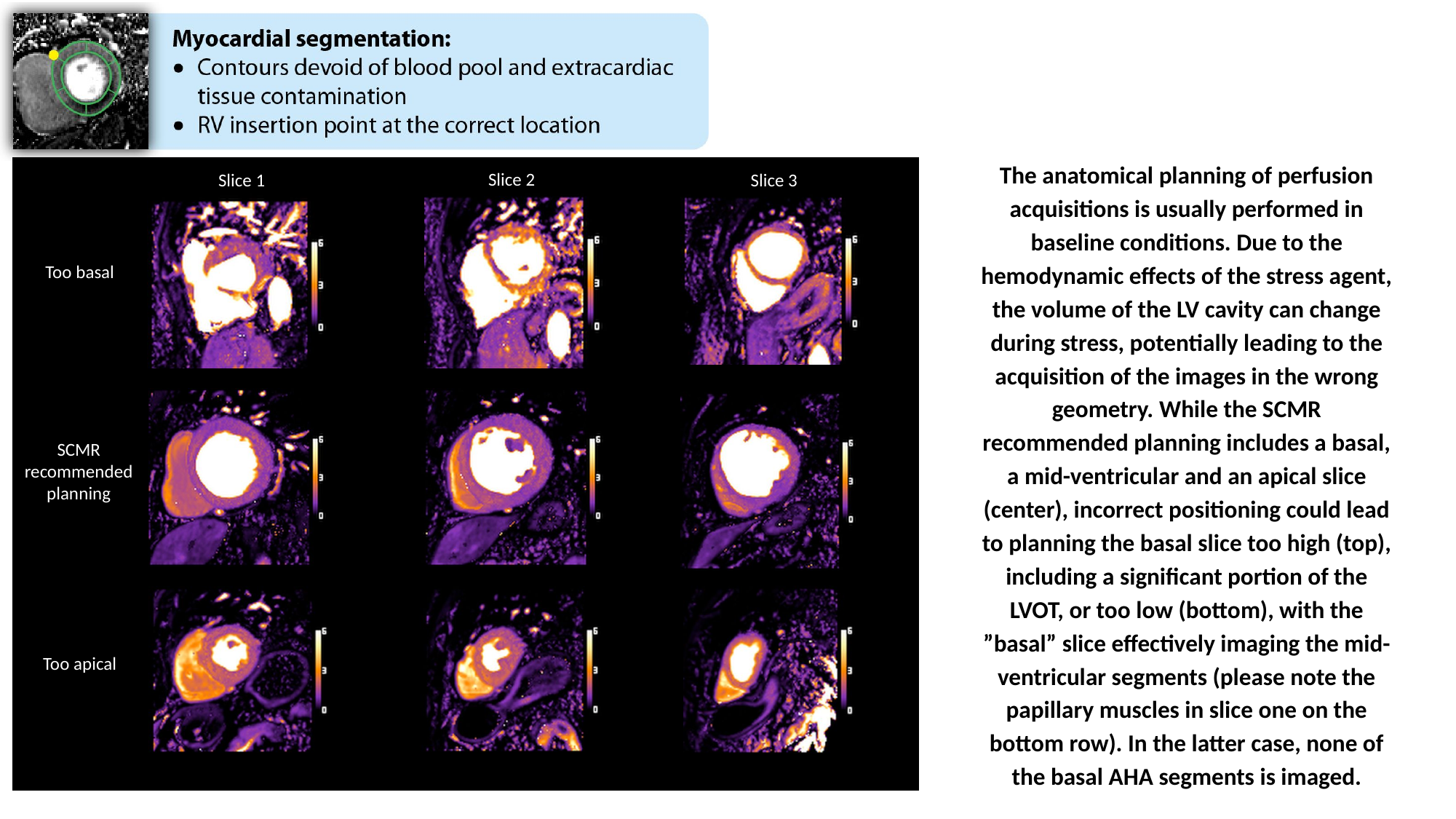

The anatomical planning of perfusion acquisitions is usually performed in baseline conditions. Due to the hemodynamic effects of the stress agent, the volume of the LV cavity can change during stress, potentially leading to the acquisition of the images in the wrong geometry. While the SCMR recommended planning includes a basal, a mid-ventricular and an apical slice (center), incorrect positioning could lead to planning the basal slice too high (top), including a significant portion of the LVOT, or too low (bottom), with the ”basal” slice effectively imaging the mid-ventricular segments (please note the papillary muscles in slice one on the bottom row). In the latter case, none of the basal AHA segments is imaged.
Slice 2
Slice 1
Slice 3
Too basal
SCMR
recommended
planning
Too apical

## Slide 9
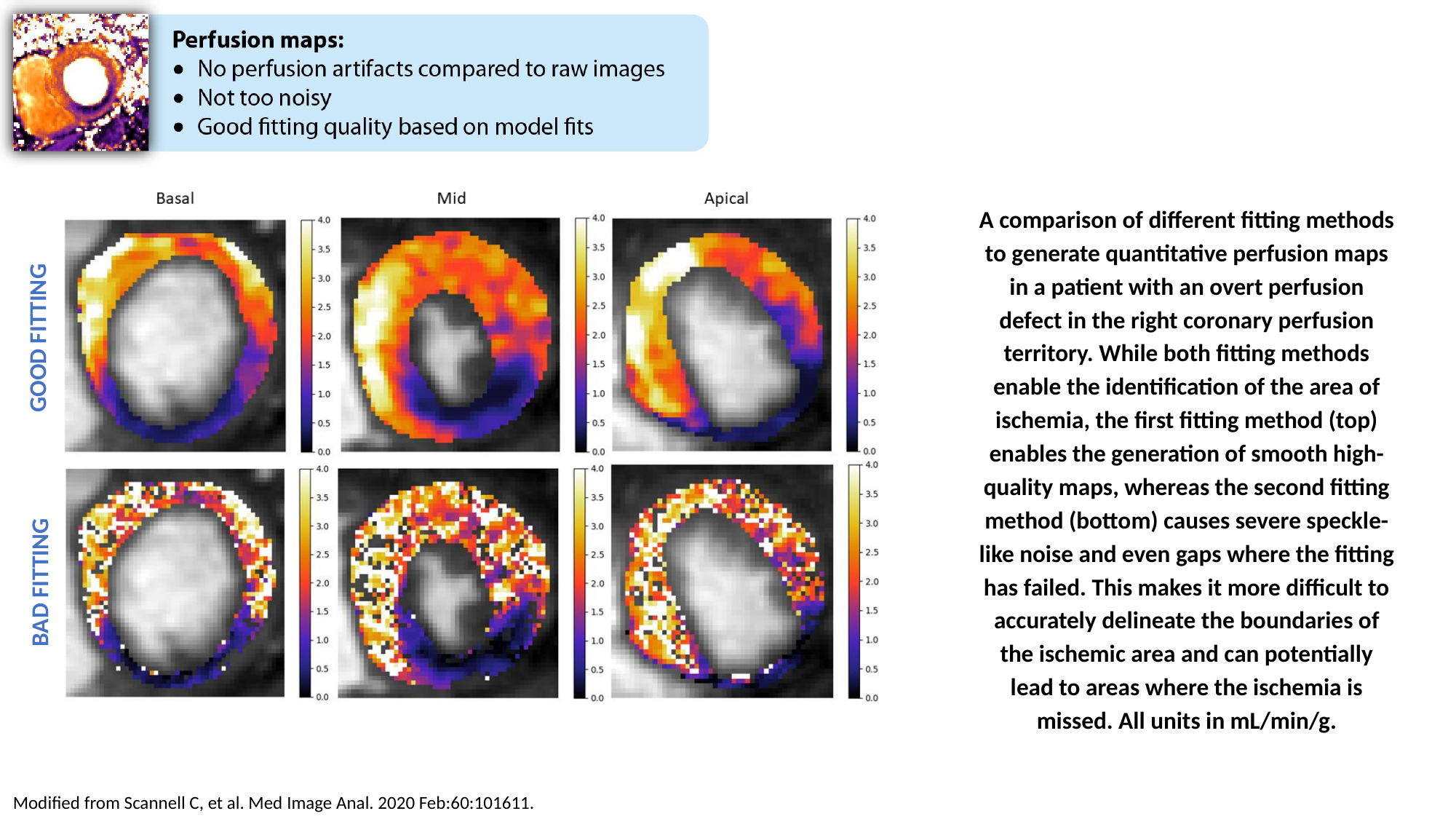

A comparison of different fitting methods to generate quantitative perfusion maps in a patient with an overt perfusion defect in the right coronary perfusion territory. While both fitting methods enable the identification of the area of ischemia, the first fitting method (top) enables the generation of smooth high-quality maps, whereas the second fitting method (bottom) causes severe speckle-like noise and even gaps where the fitting has failed. This makes it more difficult to accurately delineate the boundaries of the ischemic area and can potentially lead to areas where the ischemia is missed. All units in mL/min/g.
GOOD FITTING
BAD FITTING
Modified from Scannell C, et al. Med Image Anal. 2020 Feb:60:101611.
